# High *Q* Hybrid Mie–Plasmonic Resonances in van der Waals Nanoantennas on Gold Substrate

**DOI:** 10.1021/acsnano.4c02178

**Published:** 2024-06-13

**Authors:** Sam A. Randerson, Panaiot G. Zotev, Xuerong Hu, Alexander J. Knight, Yadong Wang, Sharada Nagarkar, Dominic Hensman, Yue Wang, Alexander I. Tartakovskii

**Affiliations:** † Department of Physics and Astronomy, University of Sheffield, Sheffield S3 7RH, U.K.; ‡ Department of Physics, School of Physics, Engineering and Technology, University of York, York YO10 5DD, U.K.

**Keywords:** van der Waals materials, transition-metal dichalcogenides, nanophotonics, Mie–plasmonic resonances, strong coupling, bound state in the continuum, Purcell enhancement

## Abstract

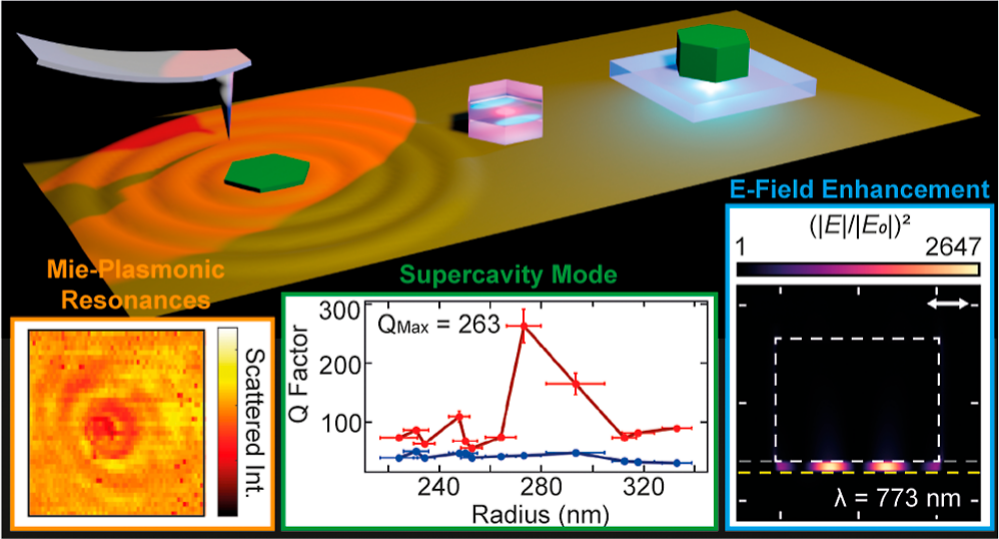

Dielectric nanoresonators have been shown to circumvent the heavy optical losses associated with plasmonic devices; however, they suffer from less confined resonances. By constructing a hybrid system of both dielectric and metallic materials, one can retain low losses, while achieving stronger mode confinement. Here, we use a high refractive index multilayer transition-metal dichalcogenide WS_2_ exfoliated on gold to fabricate and optically characterize a hybrid nanoantenna-on-gold system. We experimentally observe a hybridization of Mie resonances, Fabry–Perot modes, and surface plasmon-polaritons launched from the nanoantennas into the substrate. We measure the experimental quality factors of hybridized Mie–plasmonic (MP) modes to be up to 33 times that of standard Mie resonances in the nanoantennas on silica. We then tune the nanoantenna geometries to observe signatures of a supercavity mode with a further increased *Q* factor of over 260 in experiment. We show that this quasi-bound state in the continuum results from strong coupling between a Mie resonance and Fabry–Perot-plasmonic mode in the vicinity of the higher-order anapole condition. We further simulate WS_2_ nanoantennas on gold with a 5 nm thick hBN spacer in between. By placing a dipole within this spacer, we calculate the overall light extraction enhancement of over 10^7^, resulting from the strong, subwavelength confinement of the incident light, a Purcell factor of over 700, and high directivity of the emitted light of up to 50%. We thus show that multilayer TMDs can be used to realize simple-to-fabricate, hybrid dielectric-on-metal nanophotonic devices granting access to high-*Q*, strongly confined, MP resonances, along with a large enhancement for emitters in the TMD–gold gap.

## Introduction

In the past decade, transition-metal dichalcogenide (TMD) monolayers have attracted a large research effort owing to their direct band gap transition and useful optoelectronic properties,^1^ rendering the bulk material largely overlooked. More recently, nanoresonators utilizing bulk TMDs to host Mie resonances have gained interest; however, these studies primarily focus on fabricating such structures on low refractive index dielectric substrates such as SiO_2_.^2−8^ A hybrid TMD-nanoantenna-on-metal system has not been thoroughly explored experimentally, with the previous work being focused on different structures such as TMD gratings on gold,^9^ metallic nanoparticle-on-TMD monolayer systems,^10,11^ or hybrid silicon–metal-based structures.^12−17^ For example, Yang et al. numerically analyzed dielectric nanoantennas situated several nanometers above a silver substrate.^15^ Their work showed hybrid dielectric–plasmonic modes^15,18^ with quality (*Q*) factors up to ∼10^3^ and Purcell enhancements of >5000, along with strong electric field confinement in the nanoantenna–substrate gap. Further to this, Dmitriev et al. fabricated silicon nanorings on gold with a layer of embedded quantum emitters between them.^17^ They observed Mie resonances from the nanoring in dark field spectroscopy, along with strong directionality of the coupled emitters normal to the substrate with a fluorescence enhancement of over 650.

TMDs, however, present an attractive alternative to silicon-based nanophotonics^19^ for generating strong mode confinement owing to their higher refractive indices^20^ while also having negligible absorption over large parts of the visible wavelength range.^21^ Additionally, one can achieve the high-crystalline quality of thin films (from monolayer up to ∼500 nm) required for the fabrication of nanoantennas, through simple exfoliation from bulk TMDs.^22^ Furthermore, they can be exfoliated onto a range of other materials owing to their inherent van der Waals attractive forces.^1^ This avoids difficulties with material bonding as well as lattice matching requirements and growth in molecular beam epitaxy chambers, which are associated with fabricating nanostructures from other conventional dielectrics such as GaAs and GaP.^23^ Use of TMDs thus opens exciting additional possibilities in the design and fabrication of hybrid dielectric–metallic structures of a variety of thicknesses, enabling the advanced control of photonic and plasmonic resonances on the nanoscale.^24^

In this work, we provide detailed insights into the physics of hybrid dielectric–metallic nanophotonic systems that can host high-quality factor modes not seen in purely dielectric Mie resonators. To do this, we first simulate a WS_2_ nanoantenna suspended in a vacuum using the finite-difference time-domain (FDTD) method. We gradually move the nanoantenna closer to a gold substrate until we observe a Fano-line shape; hybrid Mie–plasmonic (MP) modes^25^ appear in the scattering spectra with strongly enhanced *Q* factors compared to Lorentzian-line shape Mie modes in WS_2_ nanoantennas on SiO_2_.

We then fabricate WS_2_ nanoantennas on a gold substrate and characterize their optical response with experimental dark field spectroscopy, which agrees well with simulations. We carefully examine all the resonances within such devices and observe dramatic changes in the mode structure compared to that of nanoantennas on silica, with improved *Q* factors, in agreement with previous work.^24^ We demonstrate the presence of hybrid MP modes in experiment that can be easily tuned to different wavelengths by changing the nanoantenna geometries. Such hybridized modes exhibit experimental *Q* factors up to 165, a factor of 33 times higher than Mie modes previously measured in the nanoantennas on silica,^24^ highlighting the potential applications in switching and sensing.^26−29^ MP modes are investigated in more detail through the simulation of their electric field distributions and experimental scattering-type scanning near-field optical microscopy (s-SNOM), confirming that such hybrid modes can enhance surface plasmon-polaritons (SPPs) launched into gold by illuminated nanoantennas.

We further explore a strong mode coupling between a Fabry–Perot-plasmonic (FPP) mode and a Mie resonance in the vicinity of the higher-order anapole condition within a WS_2_ nanoantenna on a gold substrate in both experiment and simulation. Such resonances can be tuned to realize an anticrossing in the scattering spectra, with an experimental minimum energy splitting of 48 ± 5 meV. At the point of anticrossing, we observe signatures of a highly confined supercavity mode,^30^ with a *Q* factor of 263 ± 28, resulting from destructive interference of the two modes outside of the hybrid nanoantenna structure. This offers a simple-to-fabricate solution for realizing a supercavity mode in finite-sized nanophotonic devices with applications in enhancing nonlinear effects.^31^

Finally, we highlight potential applications of the hybrid dielectric–metallic nanophotonics platform we study, by modeling WS_2_ nanoantennas on top of 5 nm thick layers of hBN attached to a gold substrate. With this device, we numerically achieve a strong electric field enhancement exceeding 2600 in the hBN spacer between the nanoantenna and gold. From this, we calculate Purcell enhancements of over 700 for a dipole polarized normal to the substrate within the hBN and light extraction efficiencies as high as 50% through a numerical aperture of 0.64. These factors combined yield an overall enhancement factor of over 10^7^, highlighting the potential for strongly enhancing the emission of coupled single-photon emitters (SPEs) or interlayer and moiré excitons^32,33^ in TMD heterostructures placed within the gap.

## Results

### Introducing the Gold Substrate

**Figure 1:**
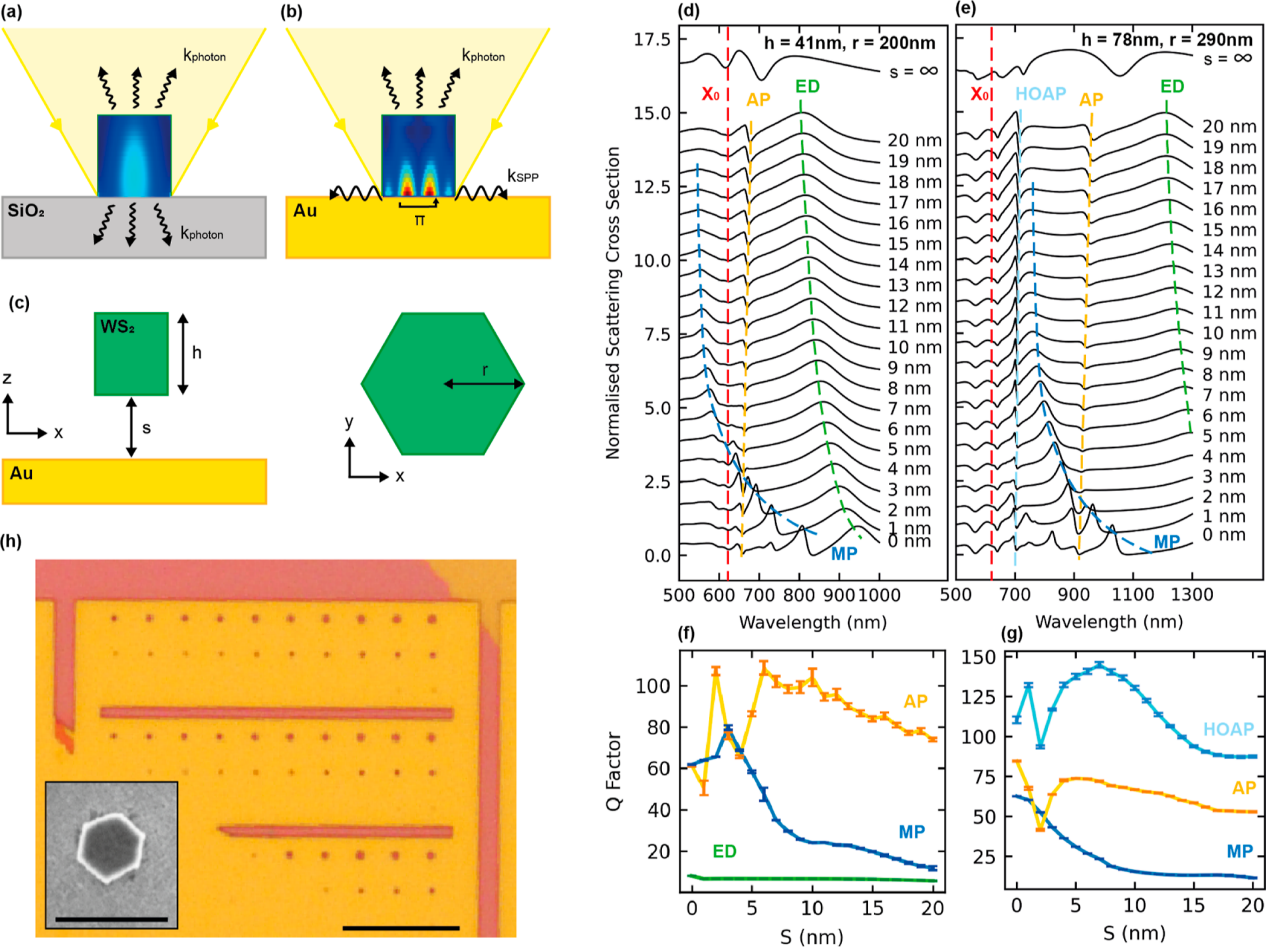
WS_2_ nanoantennas from vacuum to gold simulations. (a,b) Illustrations of WS_2_ nanoantennas on SiO_2_ and gold substrates, respectively. Inside the nanoantennas are example electric field distributions for both cases. *k*_photon_ and *k*_SPP_ correspond to the wavevector of scattered photons and guided SPPs, respectively. (c) Schematic of the structure considered in the mode tracking simulations. *h* and *r* correspond to nanoantenna height and radius, respectively. *s* corresponds to the distance between the bottom surface of the nanoantenna and top surface of the gold substrate. (d,e) Simulated normalized scattering cross section of WS_2_ nanoantennas with increasing distance from a gold substrate. Geometries are *h* = 41 nm, *r* = 200 nm, and *h* = 78 nm, *r* = 290 nm, respectively. *X*_0_ corresponds to the WS_2_ exciton, ED corresponds to the ED mode, MP corresponds to the MP mode, AP and HOAP correspond to the anapole and higher-order anapole, respectively. (f,g) *Q* factors of each simulated resonance as a function of *s*. AP, MP, and HOAP are fitted with a Fano curve, and ED mode fitted with a Lorentzian. Error bars correspond to error in the fitting. (h) Optical image of fabricated nanoantennas arranged into arrays of 30 with increasing radii. Smaller nanoantennas not visible. Inset shows a scanning electron microscopy (SEM) image illustrating the hexagonal shape of the nanoantennas. Scale bars are 10 μm for the optical image and 1 μm for the SEM image.

By considering an all-dielectric, hexagonal WS_2_ nanoantenna on a SiO_2_ system as illustrated in Figure 1a and a hybrid dielectric–metallic WS_2_ nanoantenna on a gold system as in Figure 1b, we note three important differences. The first being that incident light is reflected much more strongly from a metallic substrate than a dielectric. Therefore, we expect any resonances present in the nanoantennas to increase in quality factor owing to the gold. Second, the reflection at the gold–TMD boundary will introduce a π phase shift of light, hence doubling the effective mode volume. This means that more resonances of larger wavelength can be confined to the same volume compared to nanoantennas with a dielectric substrate. Finally, we predict there to be a plasmonic resonance effect owing to the free electrons in gold and the subwavelength confinement of light from the nanoantennas.

We first strive to understand exactly how the metallic substrate changes the mode structure of the nanoantenna system, by simulating a WS_2_ nanoantenna of fixed geometry moving toward a gold substrate in 1 nm steps. With each step, we calculate the scattering spectrum from the whole system, allowing us to incrementally visualize how the modes shift and how additional modes form during the transition between an all-dielectric and a hybrid dielectric–metallic regime. A schematic of the structure is illustrated in Figure 1c, where the distance between the nanoantenna and gold substrate *s* is reduced from 20 to 0 nm. We used the FDTD method to calculate the scattering cross sections shown in Figure 1d,e, which correspond to single hexagonal nanoantennas (monomers) of heights 41 and 78 nm and radii 200 and 290 nm, respectively. The anisotropy of WS_2_ was taken into account by using experimentally measured in- and out-of-plane refractive indices as a function of wavelength,^24^ as illustrated in Supporting Information Note 1. In order to first characterize the Mie resonances within the nanoantennas in a vacuum, we performed rigorous multipole expansions of the scattered light using the open-source software MENP.^34^ Full details of the expansion are provided in Supporting Information Note 2, where we calculate the partial scattering cross sections attributed to each individual Mie mode and assign them to their respective peaks in the overall scattering spectra. Using a long-wavelength approximation,^34^ radiationless anapole conditions^35,36^ can also be identified. This was carried out for a monomer simulated in a vacuum, as a homogeneous environment is necessary for the expansion. We note that no significant difference in the mode structure was seen for WS_2_ nanoantennas on a SiO_2_ substrate. The spectral positions of the resonances were then tracked from the nanoantenna in a vacuum case to the nanoantenna on a gold case, as shown by the dashed lines in Figure 1d,e. In addition to the modes identified in a nanoantenna in a vacuum using the exact Mie expansion and the long-wavelength approximation, in the proximity of the gold substrate, we observe additional spectral features as described below.

The green dashed lines in Figure 1d,e follow the peak scattering of the electric dipole (ED) mode as *s* is decreased from 20 to 0 nm. The peak red-shifts by 140 nm and narrows for the nanoantennas in Figure 1d (*h* = 41 nm, *r* = 200 nm), therefore increasing the quality factor from 6 to 8 as shown in Figure 1f. The yellow dashed line corresponds to the anapole condition (AP). While not an eigenstate of the system itself like the Mie modes are, the anapole can be visualized as a destructive interference of the different components of the ED moment (as explained further in Supporting Information Note 2),^35−37^ causing the suppression of the scattering in the far-field.^38^ In a similar way, higher-order anapoles (HOAP) can be observed and identified in taller nanoantennas, as shown in Figure 1e. In contrast to the ED peak, the AP exhibits a small blue shift with decreasing *s*. We attribute the shifts of the ED peak and AP to the differing coupling strength with their mirror images owing to the gold substrate. A more detailed analysis of the electric field distributions of such modes will be considered later in this study. The di2p in scattering at a constant wavelength of 625 nm is due to WS_2_ excitonic absorption (red dashed line). Interestingly, a resonance peak not seen in purely dielectric systems emerges in the spectra when *s* is reduced to the order of 10 nm. We name this mode MP,^39^ as it is a hybridization of Mie modes within the nanoantenna and plasmons in gold. This peak is much sharper than ED and red-shifts more strongly by 159 nm from *s* = 4 nm to *s* = 0 nm for the *h* = 41 nm nanoantenna (Figure 1d). A similar behavior is also seen for the MP mode of the *h* = 78 nm nanoantenna in Figure 1e. An enlarged plot of the MP mode evolution is shown in Supporting Information Note 3 for clarity, where an avoided crossing with the AP and exciton is also visible, suggesting strong coupling between this mode and the WS_2_ exciton.^2,40^

For each spectrum in Figure 1d,e, we fit the various peaks using a Fano curve for the AP, HOAP, and MP modes and a Lorentzian for the ED mode. The rationale behind such choice of curve fitting will be explained later in this study, with the example fits displayed in Supporting Information Note 4. We then extracted quality factors for each resonance by dividing the peak position by its respective line width, as shown in Figure 1f,g. We calculate a quality factor of 60 for the hybrid MP mode at *s* = 0 nm, an order of magnitude higher than that of the ED mode, as shown in Figure 1f. Furthermore, we observe from Figure 1g that both AP and HOAP increase in *Q* factor by approximately 1.5 times as the nanoantennas are moved closer to the gold substrate, highlighting the immediate advantage of using a metallic substrate over a dielectric. The *Q* factors show apparent dips and discontinuities at smaller values of *s*, which can be explained by the spectral proximity of AP and HOAP to other modes or the WS_2_ exciton, where we expect some hybridization. Such interference of multiple photonic modes will be explored in more detail later in this study.

### Dark Field Spectroscopy of Fabricated WS_2_ Nanoantennas on Gold

We realized WS_2_ nanoantennas on a gold substrate using well-established nanofabrication techniques including mechanical exfoliation of bulk WS_2_ onto gold substrates, spinning of a positive resist, electron beam lithography (EBL) patterning, and reactive ion etching (RIE) (see Methods for full details). An isotropic etching recipe with SF_6_ gas yielded nanoantennas of specified radii with hexagonal cross sections, owing to a faster etching of the armchair axis of the crystal.^5,20,41^ In addition, the gold substrate acted as a natural etch stop, thus producing nanoantennas flat to the gold substrate, rather than on a pedestal of substrate material like with SiO_2_ which is etched by SF_6_. Figure 1h shows both optical and SEM images of the finalized nanoantennas.

**Figure 2:**
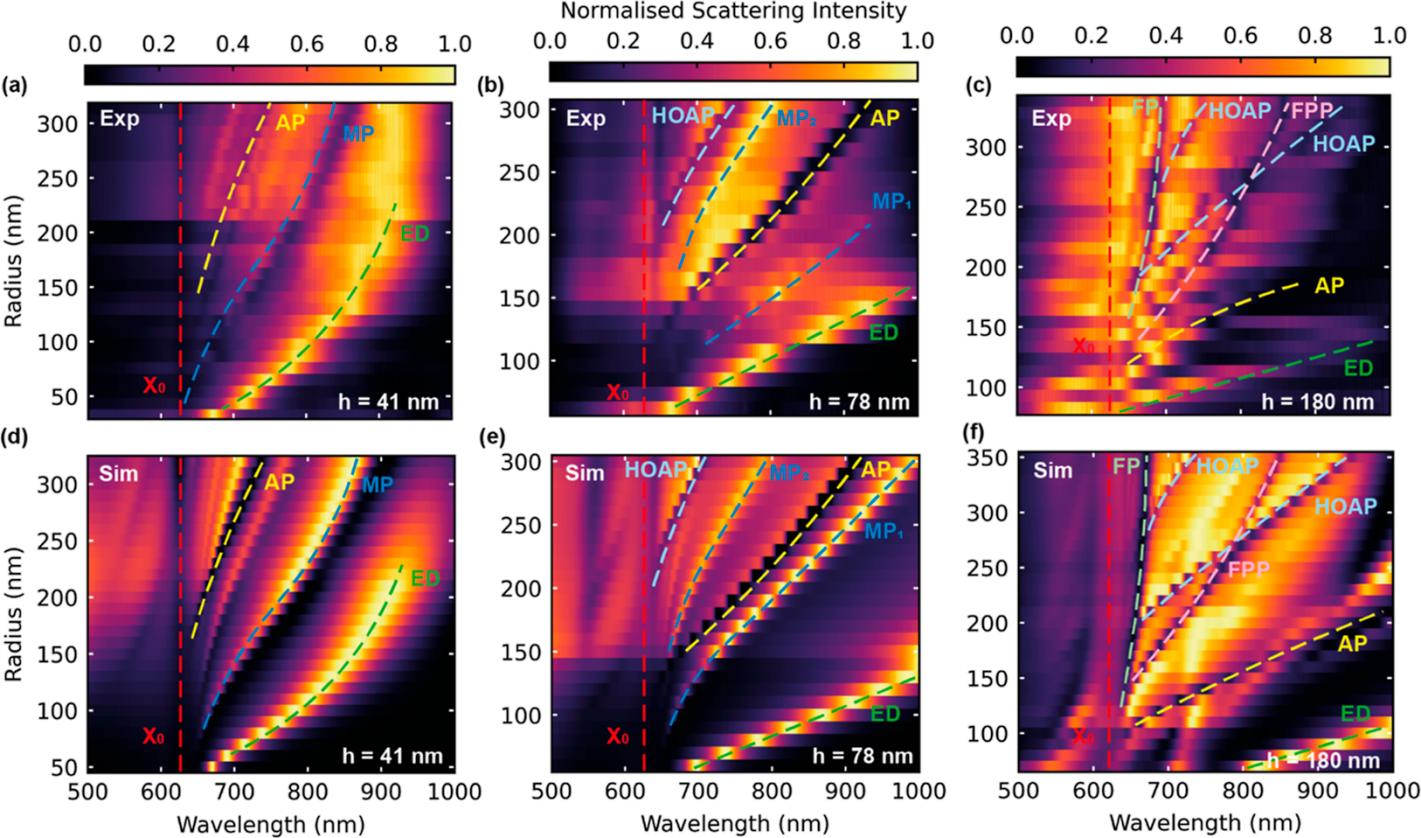
Optical characterization of WS_2_ nanoantennas on gold. (a–c) Normalized scattering cross section from experimental dark field spectroscopy of nanoantennas on gold of heights 41, 78, and 180 nm, respectively. (d–f) Simulated normalized scattering cross section of WS_2_ nanoantennas for the same geometries as in experiment with the refractive index data from Zotev et al.^24^ Left and central columns show data corresponding to single pillars (monomers), while right column corresponds to double pillars (dimers) with a separation of 475 nm. ED corresponds to the ED mode, and AP and HOAP correspond to the anapole and higher-order anapole states, respectively. MP, FP, and FPP correspond to MP, FP, and FPP modes, respectively. *X*_0_ represents the WS_2_ exciton.

In order to optically characterize our fabricated samples, we carried out dark field spectroscopy on individual WS_2_ nanoantennas on gold. We measured three different heights of nanoantennas (41, 78, and 180 nm, as measured by atomic force microscopy (AFM)) with a range of radii for each and plotted the normalized scattering intensity in Figure 2a–c. Panels d–f show the simulated scattering cross sections for the same range of heights and radii using the FDTD method, exhibiting very good agreement. We attribute any slight discrepancies between the calculated and measured spectra to fabrication imperfections and error in the experimentally measured refractive index data^24^ used for simulation. Note that panels c–f correspond to double nanoantennas (dimers) with a gap between them on the order of 500 nm. We do see a significant change in the scattering intensity profiles between monomers and dimers for such large gap sizes.^5^

The mode structure becomes increasingly complex as the nanoantenna height is increased. In the simplest case of monomers with height 41 nm (Figure 2a,d), we observe a variety of maxima and minima in the scattering spectra that red-shift with increasing radius corresponding to the ED, AP, and MP modes. The dip in scattering at a constant wavelength of 625 nm is due to absorption from the WS_2_ exciton, where we observe avoided crossings with the Mie modes as previously reported.^2^ As the height is increased to 78 nm (Figure 2b,e), we introduce the HOAP and an additional MP mode, and we denote these MP_1_ and MP_2_. We extracted the quality factor of the ED and MP modes in experiment for WS_2_ nanoantennas on gold of height 78 nm from Figure 2b. A Lorentzian function was fitted to the ED mode and a Fano curve for the MP modes to calculate the maximum *Q* factors. In order to avoid any strong light–matter interaction which could impact measured line widths, we only considered modes >100 nm from the WS_2_ exciton, far from any Rabi splitting. We measured a maximum *Q* factor of 13 for the ED mode and 72 for the MP_2_ mode. The MP_1_ mode reaches a significantly higher maximum *Q* factor of 165. We then compared these *Q* factors to those previously measured in experiment for Mie resonances in WS_2_ nanoantennas of similar sizes but on a SiO_2_ substrate, reaching a maximum *Q* factor of 5 for the ED mode.^24^ This yields a 33-fold increase in the maximum achievable *Q* factor when switching to a metallic substrate, demonstrating the strong confinement that can be achieved through our hybrid nanoantenna-on-metal system compared to purely dielectric nanoantennas.

The quality factors of our simulated scattering spectra agree well with experiment for the ED and MP_2_ modes. Only the *Q* factor of MP_1_ is not well reproduced in simulation. We attribute this to the discrepancies in the refractive index data used, simulation mesh size constraints, and imperfections in the fabricated nanoantennas.

When the nanoantenna height is increased to 180 nm as in Figure 2c,f, the mode structure becomes much more complex. Not only do we see anapole and higher-order anapole states, but similar to previous simulations^30,42−45^ and experiments^46−49^ of various dielectric nanostructures, we observe a Fabry–Perot (FP) mode trapped between the TMD–gold interface at the bottom of the nanoantenna and the TMD–air interface at the top. This mode shifts very little with changing radius, as would be expected from a vertically propagating FP mode. A more rigorous definition with comparison to the FP mode theory is provided in Supporting Information Note 5. We also observe a FPP mode^49^ having a distinctly different electric field distribution from the FP mode, with maxima in the proximity of the WS_2_/gold interface, as shown in more detail in Supporting Information Note 8. We find that this FPP mode hybridizes with Mie modes in the vicinity of the HOAP condition. This can be seen by a characteristic anticrossing in both the experimental and simulated scattering spectra of the dimer nanoantennas plotted in Figure 2c,f, with radii between 270 and 280 nm at a wavelength of around 800 nm, and is investigated in more detail later in this study.

### MP Mode Characterization

**Figure 3:**
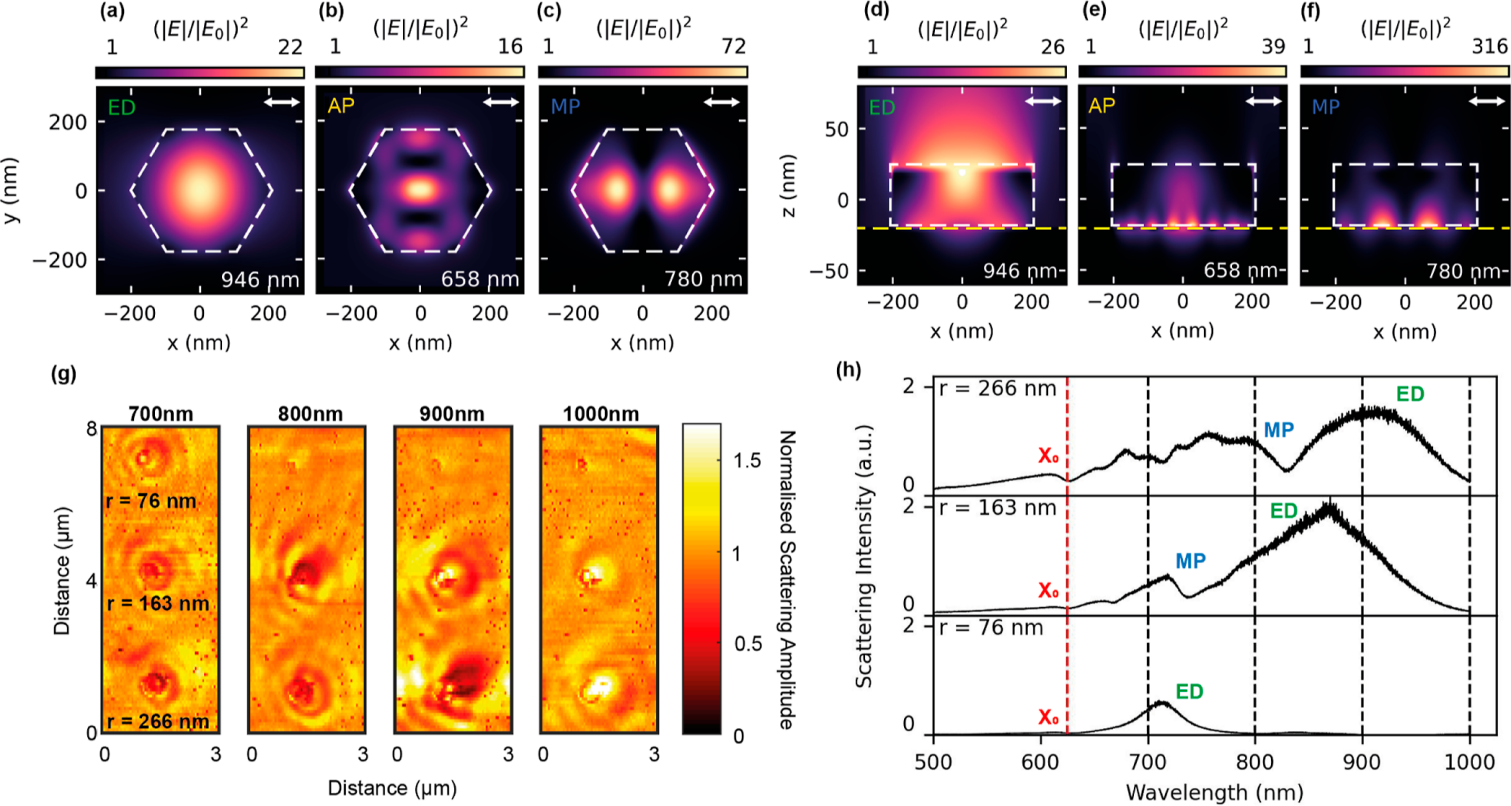
Characterization of MP resonances in WS_2_ nanoantennas on gold. (a–f) Electric field distributions for different resonant modes within a nanoantenna of height 41 nm and radius 200 nm for *s* = 0 nm. Both the *xy* (left) and *xz* (right) perspectives show a slice through the middle of the nanoantennas in the corresponding planes. White and yellow dashed lines represent the edges of the nanoantennas and surface of gold, respectively. Bottom right value corresponds to the incident wavelength. (g) Experimental s-SNOM scattering amplitude data for three WS_2_ nanoantennas on a gold substrate for varying incident wavelengths as labeled above each image. All data normalized to the scattering amplitude from an area of gold free from SPP effects. Nanoantenna heights are all 41 nm with radii 76 nm (top), 163 nm (middle), and 266 nm (bottom) for comparison. (h) Normalized experimental dark field scattering intensity for the three WS_2_ nanoantennas on gold from (g). Black vertical dashed lines correspond to excitation wavelengths used in the s-SNOM measurements. Red dashed line represents the WS_2_ exciton (*X*_0_). MP and ED correspond to the MP and ED modes, respectively.

The mode structure of WS_2_ nanoantennas in a vacuum and on low-index substrates is very different compared to the case with a gold substrate, as seen from Figure 1d,e (also see Supporting Information Note 6 for a full comparison of substrates over a range of nanoantenna radii). In order to gain further insights into the origin of the modes observed in WS_2_ nanoantennas on gold, we simulated the electric field distributions within a monomer of a fixed geometry (*r* = 200 nm, *h* = 41 nm) positioned on a gold substrate in Figure 3a–f. By first considering the ED mode in the *xy* plane (Figure 3a), we see one prominent, symmetrical field maxima in the center of the nanoantenna as expected. This is similar to the case of the ED mode in a nanoantenna in a vacuum (see Supporting Information Note 7) but less confined at the nanoantenna vertices. However, by changing the perspective to the *xz* plane as in Figure 3d, we observe that the central lobe protrudes upward and out of the top of the nanoantenna with the introduction of the gold substrate, making the mode volume larger compared to that in a vacuum (see Supporting Information Note 7). We therefore attribute the red shift of the ED mode with decreasing *s* from Figure 1d,e to this increased mode volume stemming from the interaction of the mode with its mirror image.

By considering the electric field profile of the anapole shown in Figure 3b, we see a characteristic field distribution in the *xy* plane.^38^ However, in the *xz* plane (Figure 3e), the central field maxima extends slightly less out of the bottom of the nanoantenna compared to the case of a nanoantenna in a vacuum (see Supporting Information Note 7) owing to the gold substrate and remains localized mostly within the structure, unlike the ED mode in Figure 3d. The AP mode likely has a different coupling strength to its mirror image compared to the ED mode and thus causes the blue shift with decreasing *s* shown in Figure 1d,e. Lastly, we simulated the field distribution of the MP mode as in Figure 3c,f. We observe a pattern not seen in previously all-dielectric nanoantenna systems, where the electric field in the *xz* plane is very strongly localized to the gold–TMD boundary. This suggests that there is a contribution from plasmons, hence the naming MP. Interestingly, the MP mode takes a Fano line shape in the spectra as opposed to a Lorentzian, which suggests an interference between a discrete state and a continuum of states.^28,29,50^ Both the AP and HOAP modes also exhibit Fano line shapes, whereas they can be described by a Lorentzian function for the case of nanoantennas on a dielectric substrate or surrounded by vacuum, as shown in Supporting Information Note 6. Scattering spectra for identical geometries of WS_2_ nanoantennas on gold, SiO_2_, and in a vacuum are presented for further confirmation of the different line shapes with different substrates, suggesting that the gold substrate introduces an interference between a continuum (i.e., plasmons) and a discrete state (a Mie mode within the nanoantennas). Furthermore, the MP mode is not observed in the cases with nanoantennas on SiO_2_ or in a vacuum and neither does it appear in previous experimental or numerical dark field studies of all-dielectric nanoantenna structures.^2,3,5−7^ This evidence further supports that there is a hybridization of both Mie and plasmonic modes present in our TMD nanoantenna-on-gold system.

The gold–TMD interface field localization can also be observed for the ED and AP cases. However, the relative electric field enhancement at the boundary is much weaker. This suggests that both ED and AP also hybridize with the plasmons in gold along with the MP mode but not as strongly. Plasmonic hybridization can also be observed for the FPP mode from Figure 2c,f. Supporting Information Note 8 shows electric field distributions of the FPP mode, along with the remaining FP mode and HOAP for WS_2_ nanoantennas on gold. While the FP mode exhibits vertically arranged maxima indicative of FP resonances, the FPP mode has a strong localization at the gold–TMD interface. We therefore consider this no longer a pure FP mode but a hybridization of an FP cavity and the plasmon continuum.

To further characterize the nanoantennas and verify the mode hybridization with plasmons, we performed s-SNOM on the arrays of WS_2_ nanoantennas of height 41 nm on gold, for a range of wavelengths from 700 to 1000 nm. In short, s-SNOM enables us to probe both material optical properties and localized electric fields such as those from SPPs.^51,52^ Evidence for SPPs can be observed in Figure 3g, where ripple patterns appear on the surface of gold in the s-SNOM amplitude images around the nanoantennas. These correspond to an interference between light scattered to the detector directly from the probe region and electric field contributions from the tip- and nanoantenna-launched SPPs, among other mechanisms.^51−55^ A more in-depth explanation of the principles of s-SNOM can be found in the Methods section, along with details on the various mechanisms of fringe formation in s-SNOM images from our system in Supporting Information Note 9.

From Figure 3g, we observe that the amplitude of the ripples corresponds strongly to the nanoantenna size and incident wavelength. By comparing the s-SNOM amplitude data in Figure 3g to the dark field spectra of the same nanoantennas as in Figure 3h, we see a correlation between the amplitude of the SPP ripples and peaks in the respective dark field spectra. Note that we expect a minimal shift in the resonances between s-SNOM and dark field measurements since the excitation angles are comparable at 60 and 53°, respectively. At shorter wavelengths, such as 700 nm, we observe the highest amplitude ripples from the smallest nanoantenna (*r* = 76 nm), followed by the *r* = 163 nm nanoantenna. This observation matches with our experimental dark field data, where we see a strong resonant ED mode around 700 nm for the *r* = 76 nm nanoantenna and the MP mode for the *r* = 163 nm nanoantenna. As the incident wavelength is increased to 800 nm, the *r* = 76 nm nanoantenna begins to resonate less in the s-SNOM amplitude images, and the *r* = 163 nm nanoantenna shows a stronger SPP interference pattern, which correlated to the excitation of the MP mode seen in the dark field spectra of Figure 3h. At 900 nm illumination, both the *r* = 163 and 266 nm nanoantennas exhibit strong SPP ripples, following the red shift of the ED mode in the dark field data. Finally, at 1000 nm illumination wavelength, the amplitude of the SPP interference pattern around all of the nanoantennas is much lower, corresponding to the dip in the overall scattering in the dark field spectra. Additional s-SNOM amplitude data of an array of 30 nanoantennas illuminated at various wavelengths can be found in Supporting Information Note 10, along with the corresponding phase data in Supporting Information Note 11 for further reference. These observations suggest that TMD nanoantennas on gold can both scatter light to the far-field and couple light to SPPs detectable in the near-field. We thus conclude that SPPs can be launched via the excitation of various Mie resonances, such as the ED and MP modes within the nanoantennas, hence providing further evidence for the coupling between Mie and plasmonic modes.

In Supporting Information Note 12, we show an area of the sample with pillars of resist between 25 and 35 nm in height on gold. We observe negligible optical response from the resist pillars, which can be attributed to their low refractive index of 1.49.^56^ We therefore expect tip-launched SPPs to be mostly transmitted through such structures with little reflection back to the tip. In addition, low-index nanopillars do not support well-confined photonic modes unlike the WS_2_ nanoantennas. This observation further supports our previous statement, suggesting that only the high-refractive-index WS_2_ nanoantennas can launch SPPs through the excitation of strongly confined, hybrid MP resonances.

### Supercavity Mode in WS_2_ Nanoantennas on Gold

**Figure 4:**
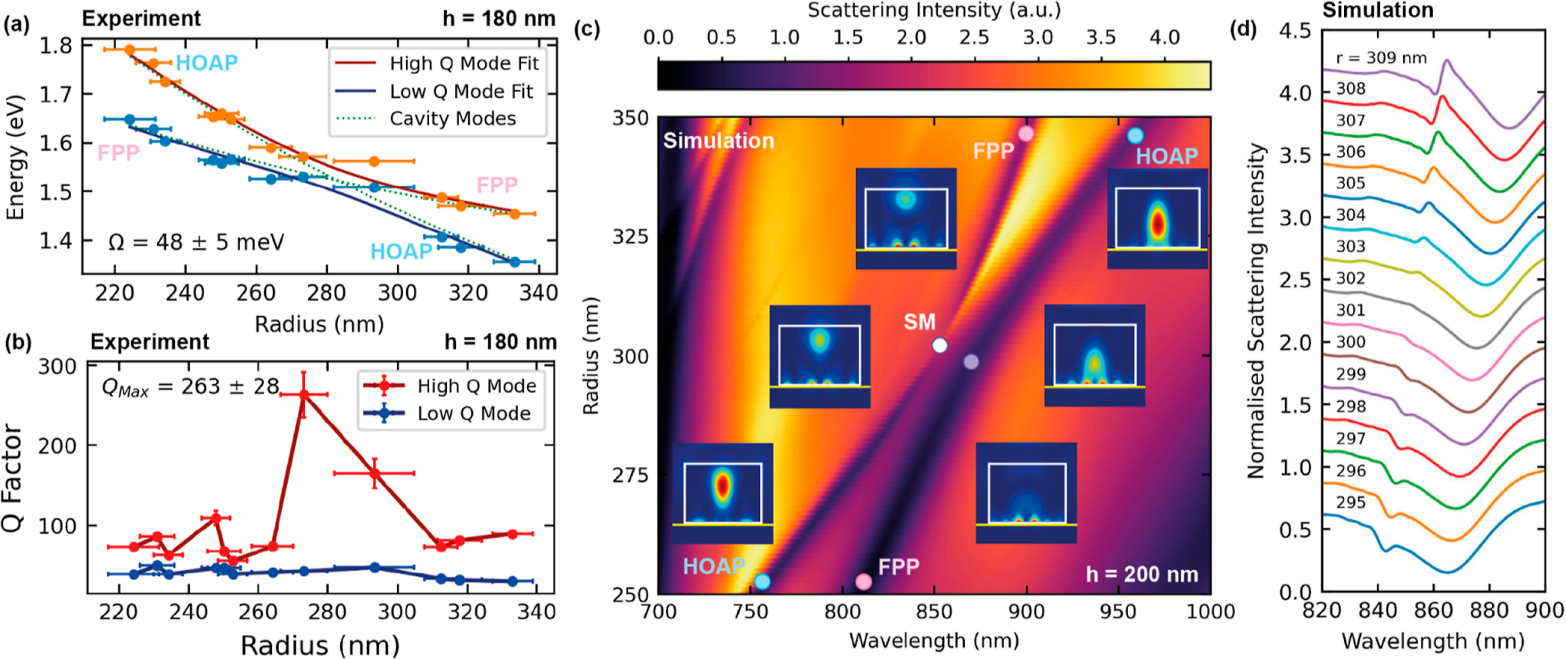
Supercavity mode (SM) characterization for WS_2_ dimer nanoantennas on a gold substrate. (a) Experimental peak center positions of the anticrossed HOAP and FPP modes fitted to a coupled oscillator model, yielding a minimum energy splitting of 48 ± 5 meV. Error bars represent uncertainty in the measured radii of the nanoantennas. The nanoantenna height is 180 nm and separation is 475 nm. Green dotted lines correspond to the uncoupled HOAP and FPP modes as obtained from the model. (b) Quality factor of the two modes with respect to radius. Error bars represent both uncertainty in the measured radii (horizontal) and error in the fits (vertical). *Q* factor calculated as the central wavelength of each peak divided by its respective full width-at half-maximum. (c) Simulated scattering spectra corresponding to optimized WS_2_ dimers of height 200 nm and the same separation over a range of radii increasing in steps of 1 nm on a gold substrate. Pink and blue circles correspond to the FPP mode and HOAP, respectively. White central circle denotes the position of the high *Q* factor SM, and the purple circle corresponds to the low *Q* factor lossy mode. Remaining insets show electric field distributions through the center of a single nanoantenna in the *xz* plane at the corresponding circles in the scattering spectra for reference. White boxes highlight the edges of the nanoantennas, and yellow lines correspond to the position of the gold substrate. (d) Waterfall of normalized simulated scattering spectra from (c) for different radii showing suppression of the high *Q* factor mode for the nanoantenna radius corresponding to the minimum wavelength splitting between the two modes (*r* = 302 nm).

In addition to the plasmon hybridization discussed previously, we observe signatures of a highly confined, nonradiating supercavity mode in both experiment and simulation by tuning the radii of taller WS_2_ nanoantennas on gold. This occurs as a result of the destructive interference of two different photonic modes outside of the nanoantenna, thus forming an extremely confined mode with a *Q* factor that, in theory, increases to infinity.^30^ This is analogous to a Friedrich–Wintgen bound state in the continuum (BIC)^57^ but for a finite-sized structure such as a nanoantenna. Experimentally and in computer simulations, we observe signs of this in the form of an anticrossing between the FPP mode^49,58^ and the minimum in the scattering identified as the HOAP condition, as seen in Figure 2c. Although the accompanying electric field distribution should involve the FPP and other Mie modes, we describe this anticrossing phenomenologically as a strong coupling between the FPP and HOAP by fitting the peak positions to a coupled oscillator model, as shown in Figure 4a.

We also note that HOAP was chosen over the lowest-order anapole condition owing to its closer spectral proximity to the FPP mode. However, we expect that strong coupling with the lowest-order anapole is also possible in further tuned versions of our structures, with similar observations in previous studies.^2,40,58^

We first fitted the HOAP and FPP peaks with a double Fano formula to account for the hybridization of the individual modes with plasmons, as detailed in Supporting Information Note 13. We then extracted the peak center positions and plotted them in terms of energy against the nanoantenna radius, as shown in Figure 4a. The error bars represent the uncertainty in the measured radii of each nanoantenna, with one data point at *r* = 293 nm showing a notably large error. We attribute this uncertainty to fabrication imperfections of this particular nanoantenna, which had a more irregular hexagonal cross section than others when imaged with SEM, thus making the determination of its radius less reliable. The error in the fitted peak energy is negligible. The peak center positions were then fitted to a coupled oscillator model, yielding the upper and lower energy branches, shown as red and blue lines, respectively. We refer to these as the high and low *Q* factor modes, respectively. The green dotted lines represent the uncoupled HOAP and FPP modes as obtained from the model. From this fitting, we extract a minimum energy splitting of 48 ± 5 meV. This is greater than the sum of the half-line widths of the uncoupled modes (34 ± 1 meV), and hence we confirm a strong mode coupling^45^ between the HOAP and FPP modes.^58^

Furthermore, we calculate the *Q* factor of each peak and plot against the nanoantenna radius in Figure 4b. While the low *Q* factor mode remains mostly constant, the high *Q* factor mode increases significantly at a radius of 273 nm. This radius corresponds to the closest point to the anticrossing in Figure 4a. We observe a maximum *Q* factor of 263 ± 28, an order of magnitude larger than that for the ED mode reported earlier in this study. This sharp increase in *Q* factor and the observation of strong mode coupling are both signatures of a supercavity mode, similar to those seen in Si nanowires^59^ and AlGaAs nanoantennas.^60^ However, owing to the confinement introduced from the gold substrate and the interaction with plasmons in our hybrid TMD nanoantenna-on-metal structure, we demonstrate a supercavity mode hosted within a volume 40 times smaller than previous dielectric nanoantennas on a SiO_2_/ITO/SiO_2_ substrate.^60^

In order to better understand the mode behavior around the anticrossing, we performed FDTD simulations of WS_2_ nanoantennas of height 200 nm on gold for a range of radii of 250–350 nm, with a much finer step in radius of 1 nm, as shown in Figure 4c. This greater height was chosen in order to red-shift the anticrossing away from other modes in the scattering spectra to aid with fitting. The anticrossing is clearly reproduced in the simulated spectra, and we extract an energy splitting of 37.5 ± 0.3 meV (see Supporting Information Note 13), agreeing with our experimental observations. We also simulated the electric field distribution within a single nanoantenna of the same dimensions as the dimer at the points marked by the colored circles in Figure 4c. Note that we found no significant differences between the scattering spectra and electric field distributions of monomer and dimer nanoantennas with such large gaps on the order of 500 nm, and the single nanoantenna electric fields are shown for clarity. Away from the point of anticrossing, the FPP mode displays clear maxima and minima in the *xz* plane, as would be expected from a FP mode confined vertically within the nanoantenna.^45^ However, we also see evidence of hybridization with plasmonic modes at the gold–WS_2_ boundary, similar to the MP modes described previously in this study. In addition, we do not observe this mode in WS_2_ nanoantennas on a SiO_2_ substrate in either simulation or experiment.^24^ We therefore attribute this resonance to a hybrid FPP mode as a result of reflections from, and SPPs at, the WS_2_–gold interface. For smaller radii, the FPP mode appears mostly plasmonic, with the field maxima near the bottom of the nanoantenna. In contrast, the HOAP field distribution is strongly localized at the center of the nanoantenna, exhibiting little hybridization with plasmons. As the radius is increased, the electric field profile of the FPP mode hybridizes with that of HOAP to form a supercavity mode labeled SM. Upon increasing the nanoantenna radius further, HOAP returns to a similar field distribution as before the anticrossing, with the central field maxima localized closer to the gold interface. However, the FPP mode field maxima is pushed up toward the top of the nanoantenna, while retaining the characteristic plasmon field distribution at the bottom.

We further investigate the suppression of the high *Q* factor mode as the nanoantenna radius is tuned. Figure 4d shows individual simulated scattering spectra from Figure 4c for a range of radii close to the anticrossing. While the low *Q* factor mode (higher wavelength) remains mostly the same, the high *Q* factor mode (lower wavelength) becomes almost invisible for a radius of 302 nm. This suppression of scattering corresponds to the point where the HOAP and FPP modes destructively interfere near perfectly, forming a highly confined resonance within the nanoantenna confirmed by the exponentially increasing *Q* factor in Supporting Information Note 13. Our simulations thus provide additional evidence to support our observation of a supercavity mode in hybrid WS_2_-on-gold nanoantennas in experiment. One can envisage applications of such high-*Q* factor modes in boosting nonlinear effects on the nanoscale, such as second harmonic generation enhancement (SHG) of a TMD monolayer^31^ coupled to WS_2_ nanoantennas on gold or further enhancement of SHG efficiency in quasi-bulk TMD pillars containing a low-symmetry interface.^24^ Other applications include quasi-BIC-enabled lasing^61^ and directionality control when considering a metasurface of nanoantennas,^62^ highlighting the potential for using nanoantennas on gold within complex integrated optical circuits.

### Purcell Enhancement of Emission between WS_2_ Nanoantennas and a Gold Substrate

**Figure 5:**
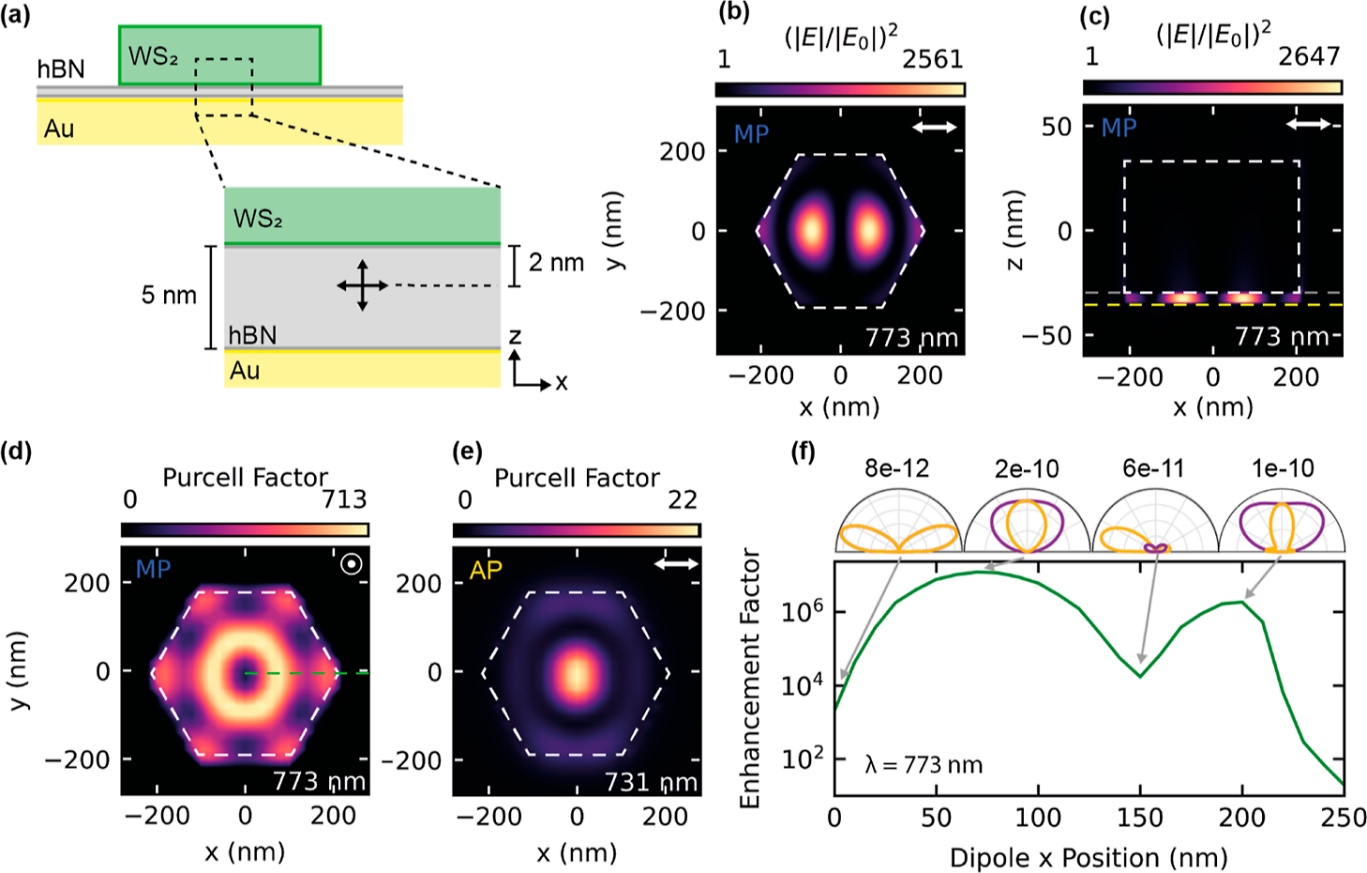
Simulated electric field and light extraction enhancement throughout an hBN layer between a WS_2_ nanoantenna and a gold substrate. (a) Schematic of the structure showing the *z* position at which the dipole was placed to simulate the Purcell factor. Crossed arrows represent the two polarizations considered. Nanoantenna of height 60 nm and radius 210 nm. (b,c) Correspond to electric field distributions in the *xy* plane within the nanoantenna–substrate gap and the *xz* plane through the center of the nanoantenna, respectively, when illuminated by a 773 nm plane wave. Dashed white lines represent the edges of the nanoantenna, yellow denotes the gold–hBN boundary, and gray corresponds to the top surface of hBN. (d,e) Maps of the maximum Purcell factor for a dipole oriented perpendicular (polarized along *z*) and parallel (polarized along *x*) to the substrate, respectively, in the same plane as (b). Dipole wavelength set to 773 and 731 nm, respectively, corresponding to the MP mode and the AP mode. (f) Total enhancement of a dipole within the hBN layer with respect to position along the dashed green line in (d) at a wavelength 773 nm. Polar plots show the far-field radiation patterns of a dipole at the marked *x* positions. Yellow and purple curves correspond to slices through the *y* = 0 and *x* = 0 planes, respectively. Values above the plots indicate the respective intensities for comparison.

The strong localization of the electric field at the TMD–metal boundary, depicted in Figure 3f, prompted further study into the Purcell enhancement of emitters at this position. We simulated the electric field distribution within an hBN layer of 5 nm thickness between a WS_2_ nanoantenna and the gold substrate, as shown in Figure 5a. hBN was chosen owing to its transparency throughout the visible wavelength range,^63^ low refractive index of 2.2,^64^ and the presence of single photon-emitting defects, radiating at various wavelengths from around 550 to 850 nm.^65−68^ Such a system bears similarities to other works on gold nanoparticle-on-mirror (NPoM) structures,^10^ however boasting several advantages. Dielectric materials offer lower losses than the solely plasmonic resonances of the gold nanoparticles; therefore, we can achieve higher *Q* factor modes and thus stronger Purcell enhancement. Furthermore, the WS_2_ nanoantennas could be fabricated experimentally with precise size, geometry, and positioning unlike the random nature of gold nanoparticle deposition. This allows a wider control over the resonances and overall greater functionality.

The simulation results are displayed in Figure 5b,c for a nanoantenna of height 60 nm and radius 210 nm, showing field distributions of similar shape to those for silicon nanoantennas above gold with a SiO_2_ spacer.^13^ The geometry of our WS_2_-nanoantenna-above-gold system was optimized for the maximum possible Purcell factor within the hBN layer over the wavelengths previously reported for hBN SPEs. We calculated a maximum electric field enhancement of 2647 within the hBN spacer at 773 nm wavelength (the MP mode);: 2 orders of magnitude higher than the maximum field within the nanoantennas for the ED mode (Figure 3d) and 1 order of magnitude higher than that of the MP mode inside a nanoantenna directly on a gold substrate (Figure 3f). We further spatially mapped the Purcell factor of a dipole emitter placed within the hBN (see Figure 5a for dipole location), mimicking a SPE . This mapping is shown in Figure 5d,e, where the dipole was oriented perpendicular (along *z*) and parallel (along *x*) to the substrate, respectively, showing a strong variation depending on both position and polarization.

From Figure 5d, we calculated a maximum Purcell factor of 713 for a dipole polarized perpendicular to the substrate at an emission wavelength of 773 nm (the MP mode), over 2 times greater than that previously reported for silicon nanoantennas above gold for the same gap size.^16^ Comparing to the dipole polarized parallel to the substrate in Figure 5e, we observed a much lower maximum Purcell factor of 22 at 731 nm (the AP). We further integrated the total emitted intensity of the dipole in all directions over a range of wavelengths from 550 to 850 nm for varying polarizations in the *xz* plane. We observed the same strong sensitivity to dipole orientation within the hBN layer, suggesting that a particular enhancement is expected in structures where the dipole is oriented vertically. Examples are hetero- and homobilayer TMDs, where interlayer excitons can be observed with electrons and holes in adjacent layers.^69^

We also consider how the directivity of a dipole at different x positions within the hBN layer will change, thus affecting the light collection efficiency through an objective lens. We define the directivity as the fraction of light captured in the far-field through a numerical aperture of 0.64 directly above the nanoantenna-hBN-gold structure, compared to the total light emitted in all directions. This forms part of the overall enhancement factor
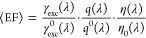
1similar to our previous work,^70^ which represents the ratio of the collected light from a dipole within the hBN spacer at a given wavelength to that of a dipole in free space. The first term of eq 1, γ_exc_/γ_exc_^0^, is the ratio of the excitation rate at a wavelength λ within the hBN spacer, compared to free space. This is proportional to the electric field intensity, , within the spacer as displayed in Figure 5b. The second term, *q*/*q*^0^, represents the quantum efficiency enhancement of the dipole at a wavelength λ. Under the assumption of a low quantum yield, this is equivalent to the radiative rate enhancement of the dipole, also known as the Purcell factor. The final term, η/η_0_, accounts for the directivity enhancement, which is the ratio of the directivity of a dipole within the hBN spacer to a dipole in a vacuum, both at wavelength λ.

The enhancement factor is presented in Figure 5f, where the wavelength is fixed at 773 nm and the dipole position in *x* is varied along the dashed green line in Figure 5d for polarization perpendicular to the substrate. Above are slices of the far-field radiation patterns in the *x* = 0 and *y* = 0 planes (purple and yellow curves) for dipole positions of particular interest. When the dipole in the hBN layer is positioned directly underneath the center of the nanoantenna (*x* = 0 nm), the Purcell factor is low as seen in Figure 5d, and most of the light is directed toward the sides of the structure as shown in Figure 5f. Therefore, very little light would be collected by an objective lens directly above the structure, and thus the overall enhancement factor is low. Note that in this case, owing to the symmetry about the center of the structure, the *x* = 0 and *y* = 0 far-field slices are identical and so overlap completely. We achieve a maximum directivity of ∼50% at *x* = 70 nm, corresponding to a high enhancement factor of 1.2 × 10^7^. As seen by the far-field radiation pattern, the dipole emits near perfectly vertically for this *x* position, and combined with a high Purcell factor and well-confined electric field, we observe a strong overall light extraction enhancement. The far-field patterns and Purcell factors explain the enhancement factors calculated at *x* = 150 and 200 nm in the same way. These results show that not only can the Purcell factor be strongly tailored depending on the dipole polarization and position within the hBN layer but also the directivity of the emitted light. Therefore, we predict that by placing systems with vertically orientated dipoles such as TMD heterostructures with interlayer excitons within the nanoantenna–gold gap, one could achieve 10^7^ times enhancement of the extracted emission. Furthermore, by changing the nanoantenna height and radius, the maximum enhancement factor can be tuned for emitters operating at different wavelengths, thus making the TMD nanoantenna–gold gap an ideal system for studying a range of low-intensity dipolar sources including SPEs, excitons, and more.

## Conclusions

In this study, we significantly built on a previous work concerning WS_2_ nanoantennas on gold by providing an in-depth analysis of the mode structure and the reasons behind why the resonances differ so much compared to that of all-dielectric nanoantenna systems, before highlighting the real applications of such devices. By simulating WS_2_ nanoantennas moving toward a gold substrate, we were able to gain insights into how the mode structure transitions from a purely dielectric to a dielectric–metallic regime, thus predicting the behavior and properties of hybrid MP modes before fabrication. Our TMD-based nanoantennas were easy to be fabricated on metals using standard nanofabrication techniques owing to their van der Waals forces acting between the TMD thin film and the substrate, with the added benefit of gold providing a natural etch stop during RIE. Once fabricated, we investigated resonant Mie modes within the nanoantennas through experimental dark field spectroscopy and observed excellent agreement with the simulation. We demonstrated that all the resonant modes identified can be tuned to different wavelengths simply by changing the nanoantenna radii and that additional, higher-order modes can be introduced by increasing the nanoantenna height. The Fano-line-shape MP modes couple to the far-field, as measured with dark field spectroscopy, and produce SPPs detectable in the near-field with s-SNOM imaging. The SPP intensities were strongly correlated to resonances in the nanoantennas, following the red shift of the modes upon increasing nanoantenna radii, further supporting our claims of hybridized MP modes. Such hybrid Fano resonances also have high *Q* factors, up to 33 times higher than Mie modes in nanoantennas placed on a low-index SiO_2_ substrate in experiment,^24^ hence enabling applications in switching and sensing.^26−29^

We further demonstrated strong mode coupling of Mie and FPP modes within WS_2_ nanoantennas on a gold substrate in experiment and simulation. We calculated a minimum energy splitting of 48 ± 5 meV, and with careful tuning of the nanoantenna geometry, we discovered signatures of a quasi-BIC supercavity mode at the point of anticrossing, including a significantly increased experimental *Q* factor of over 260 and a near-complete suppression of scattering in simulation. Here, the use of a gold substrate reveals a simple-to-fabricate method of confining a visible wavelength supercavity mode to nanometer-scale structures in experiment, with the potential applications ranging from second harmonic generation and other nonlinear effect enhancement^31^ to directional lasing.^61,62^

Finally, we observed in simulations that very strong electric field enhancement of over 2600 occurs in a nanometer scale gap between the studied WS_2_ nanoantennas and gold substrate. For a gap filled with 5 nm of hBN, we calculated light extraction efficiencies of up to 50% and a maximum Purcell factor of 713 for an emitter within the hBN polarized perpendicular to the substrate, 32 times greater than that for parallel polarization. From these values, we calculated the maximum overall enhancement of the collected light from emitters within such structures to exceed 10^7^. Hybrid WS_2_-nanoantennas-on-gold therefore introduce opportunities for strongly enhancing emitters placed within the nanoscale gap, such as SPEs in TMDs^71−82^ and hBN,^65−68^ as well as interlayer excitons in TMD bilayers^69^ and van der Waals heterostructures.^83−88^ Coupling of this system to other photonic devices such as waveguides, photonic crystals, and gratings offers near limitless combinations for using TMDs and metals together to fabricate nano-optical circuits with strong field confinement and low losses.

## Methods

### FDTD Simulations

In order to predict the behavior of light within and around our nanoantennas, the software package Lumerical from Ansys was used to perform FDTD simulations.

### Scattering Simulations

The scattering cross sections in Figure 2 were calculated by simulating WS_2_ nanoantennas of varying geometries on a semi-infinite gold substrate. To emulate dark field experiments as closely as possible, a total-field scattered-field plane wave source was used which subtracts the incident wave outside of its area of effect. This way, only the scattered light in the far-field was measured by a power monitor placed above the nanoantenna. The incident wave was set to propagate normal to the substrate and was polarized along the *x-*axis. Antisymmetric and symmetric boundary conditions were used along the *x* = 0 and *y* = 0 planes, respectively, to reduce the simulation time and memory requirements.

### Field Distributions

To visualize the electric and magnetic field distributions within and around the nanoantennas, frequency–domain field and power monitors which perform discrete Fourier transforms at chosen frequencies were used. The monitors were set as 2D surfaces through the middle of the nanoantennas used in the scattering simulations along various planes and returned the electric and magnetic field intensities normalized to the incident, vacuum wave.

### Purcell Factor Calculations

We considered a dipole emitter placed in an hBN spacer between our WS_2_ nanoantennas and a gold substrate to emulate an SPE. The wavelength was set to a range of 550–850 nm, and the orientation of the dipole rotated in the *xz* plane to consider different polarizations. The Purcell factor was then calculated as the total integrated power of the system divided by the total integrated power of the same dipole in a vacuum.

### Directivity Calculations

Near-fields from a dipole placed within the hBN spacer were extrapolated to the far-field by considering the propagating plane waves onto a hemisphere above the structure 1 m away. The far-field Poynting vector was then integrated over a solid angle characteristic of an objective lens with a numerical aperture 0.64. This value was then divided by the total power emitted in all directions to give a % of light emitted vertically from the dipole within the WS_2_-hBN-gold structure.

### Substrate Preparation

The gold substrates were fabricated using either template stripping (used in the structures measured in Figure 2a) or electron beam evaporation of roughly 150 nm of 99.99% pure gold onto a silicon wafer with a 10 nm titanium (used in the structures measured in Figure 2b) or a nickel layer (used in the structures measured in Figure 2c) to promote adhesion to gold. These had rms roughness values down to 0.7, 1.2, and 2.5 nm, respectively.

### TMD Exfoliation

WS_2_ bulk crystal from HQ-graphene was mechanically exfoliated onto the gold substrates by hand. A temperature of 105 °C was used to ensure good flake adhesion. Uniform thickness flakes of sizes 50 μm and upward were recorded for patterning.

### Electron Beam Lithography

A positive resist (ARP-9 AllResist GmbH) was first spin-coated onto the sample at 3500 rpm for 60 s before heating for 2 min at 180 °C. EBL was then performed using a Raith GmbH Voyager system at 50 kV accelerating voltage and 560 pA beam current. The pattern formed an array of circles of varying radii across the resist to cover several WS_2_ flakes.

### Reactive Ion Etching

A chemical etching recipe was used to achieve hexagonal nanoantenna geometries. Plasma etching was performed for 40 s with SF_6_ gas at 0.13 mbar pressure with a DC bias of 50 V. The armchair crystal axis of the bulk WS_2_ was preferentially etched faster than the zigzag axis, leading to 120° angles between them, forming hexagonal pillars.^41^ The gold substrate was etched much slower than WS_2_ and so acted as a natural etch stop, leaving nanoantennas on a flat gold surface, rather than on a pedestal of the substrate material. The leftover resist was then removed using a warm 1165 resist remover, before bathing in acetone, followed by IPA for 5 min, respectively. A final UV–ozone treatment of 20 min removed any residual organic debris.

### Dark Field Spectroscopy

Spectroscopy involving illuminating a sample while rejecting the reflected light and collecting only the scattered light was achieved using a Nikon LV150N microscope with a circular beam block fitted between the illumination source (tungsten halogen lamp) and the dark field objective lens (50× with 0.8 NA). The beam block used was slightly smaller than the diameter of the beam, so that the central part was discarded and only the outer ring of light entered the objective via redirection from an annular mirror. The sample was illuminated at a high oblique angle causing light to be scattered from the sample. The vertically scattered light was then collected by the objective and passed back through the hole in the annular mirror toward a 50 μm pinhole before a fiber coupler. The pinhole ensured that only light that scattered at a low angle to the normal was allowed to propagate into the 100 μm diameter core of the multimode fiber. Another fiber coupler then sent the beam into a free space path, where two achromatic lenses were used to minimize the beam diversion along the path to the spectrometer. Finally, a single achromatic lens was used to focus the beam onto the slit of a Princeton Instruments spectrometer, where the wavelength components were separated and detected by a charge-coupled device.

### s-SNOM System

Probing of the near-field scattering from our samples at the nanoscale was done using a commercial neaspec modular s-SNOM system in conjunction with a Coherent Chameleon Compact OPO-Vis pulsed laser. This technique combined a sharp AFM tip with incident radiation to strongly confine near-fields at the tip–sample interface and measure the phase and amplitude of the scattered light. The laser was aligned onto a platinum–iridium (PtIr)-coated cantilever tip (NanoWorld Arrow NCPt), with the radius of curvature less than 25 nm, using a parabolic mirror within the s-SNOM system. The beam made a 60° angle with the tip and was polarized parallel to the plane of incidence (p-polarized), to maximize the component along the tip axis. A strongly confined near-field was generated at the tip, which then interacted with the sample as it was scanned below. The background scattering signal owing to the large spot size (few microns) compared to the tip size was suppressed using neaspec’s patented pseudoheterodyne interferometry system. A reference beam with a phase modulation induced via an oscillating mirror was interfered with the scattered signal at the detector. This formed sidebands of frequency *n*Ω + *m*Δ, where Ω is the tapping frequency of the tip, and Δ is the modulation frequency of the reference mirror. The detector then locked in at the harmonics of the tapping and sideband frequencies in order to eliminate the background signal. Through using pseudoheterodyne detection, both the amplitude and phase of the scattered light were measured simultaneously.

The s-SNOM measurements in this report were demodulated at either the third (Figure 3g, Supporting Information Note 10 and 11) or fourth (Supporting Information Note 12) harmonic of Ω and the first sideband in order to reduce the background as much as possible, while still keeping a good signal-to-noise ratio.

## Supporting Information Available

In- and out-of-plane complex refractive indices for WS_2_ used in FDTD simulations; rigorous multipole expansion of Mie modes in WS_2_ nanoantennas in a vacuum; enlarged simulated scattering spectra of WS_2_ nanoantennas with changing distance above a gold substrate; example Fano and Lorentzian curve fitting to scattering spectra; FP mode theory and comparison with simulated scattering data; comparison of simulated scattering spectra of WS_2_ nanoantennas in a vacuum, on SiO_2_ and on gold; simulated electric field distributions within WS_2_ nanoantennas in a vacuum for different resonances; simulated electric field distributions within WS_2_ nanoantennas on gold for different resonances; schematic of the tip–sample interaction during s-SNOM measurements; experimental s-SNOM amplitude images of an array of WS_2_ nanoantennas on gold for incident wavelengths between 650 and 1000 nm; corresponding s-SNOM phase data; further s-SNOM images of a bare gold substrate and an array of resist pillars; double Fano fitting used to characterize the experimental and simulated supercavity mode (PDF)

## Notes

Preprint version: Randerson, S. A.; Zotev, P. G.; Hu, X.; Knight, A. J.; Wang, Y.; Nagarkar, S.; Hensman, D.; Wang, Y.; Tartakovskii, A. I. van der Waals Nanoantennas on Gold as Hosts for Hybrid MP Resonances. 2023. arXiv:2304.02537v2. arXiv. URL: https://arxiv.org/abs/2304.02537 [Accessed: 28.05.2024].
