# Supplementary material for: High Q Hybrid Mie–Plasmonic Resonances in van der Waals Nanoantennas on Gold Substrate
Source: ACS Nano. 2024 Jun 13;18(25):16208–21. doi: 10.1021/acsnano.4c02178 (PMC11210342; doi:10.1021/acsnano.4c02178)
Supplement: Supplementary file 1 — nn4c02178_si_002.pdf [file nn4c02178_si_002.pdf]

Supplementary Information for: High Q Hybrid Mie-Plasmonic Resonances in Van der Waals Nanoantennas on Gold Substrate

Sam A. Randerson,*,† Panaiot G. Zotev,† Xuerong Hu,† Alexander J. Knight,† Yadong Wang,† Sharada Nagarkar,† Dominic Hensman,† Yue Wang,‡ and Alexander I. Tartakovskii*,†

†*Department of Physics and Astronomy, University of Sheffield, Sheffield, S3 7RH, UK*

‡*Department of Physics, School of Physics, Engineering and Technology, University of York, York, YO10 5DD, UK*

E-mail: sranderson1@sheffield.ac.uk; a.tartakovskii@sheffield.ac.uk

Supplementary Note 1:

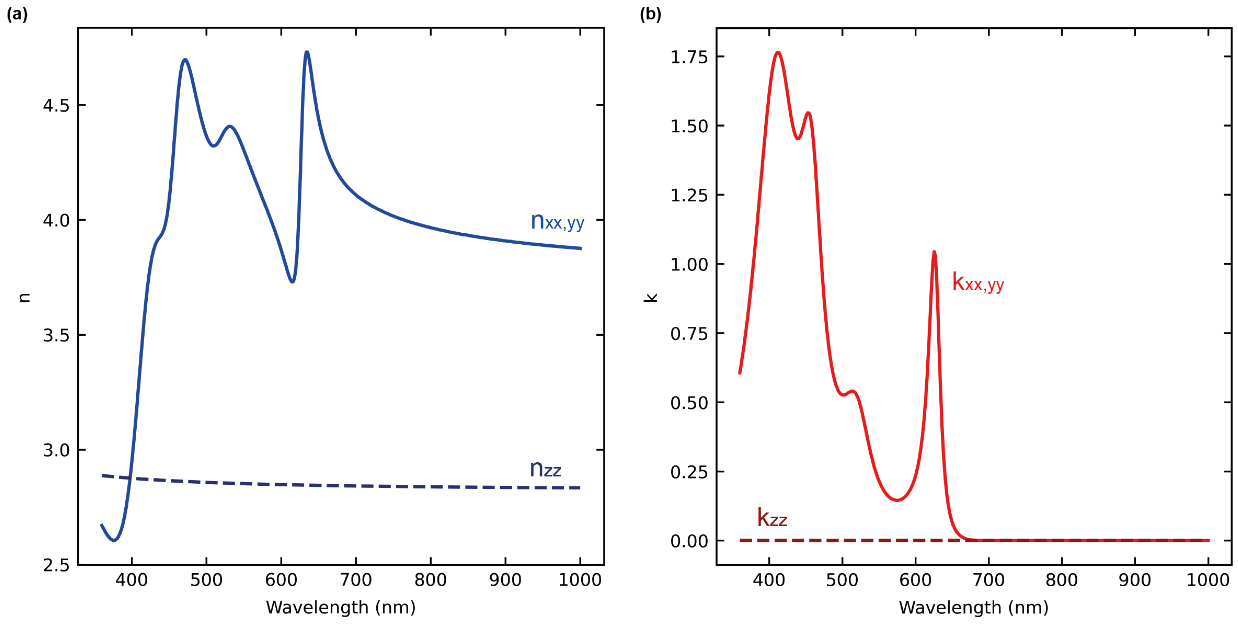

Figure S1: **Experimentally measured complex refractive index data for bulk, layered WS_2.** **(a)**, **(b)** show the real (n) and imaginary (k) parts of the refractive index respectively, for both the in-plane (solid lines) and out-of-plane (dashed lines) directions.

In order to accurately model the anisotropy of WS_2 crystal in simulation, we consider both an in-plane refractive index containing excitonic features, and an out-of-plane refractive index which is more uniform. The real and imaginary parts of the refractive index displayed in Figures S1(a) and (b) respectively, were obtained experimentally through ellipsometry of bulk WS_2.[1] We then import these data into Lumerical finite-difference time domain (FDTD), which is able to simulate the optical response of anisotropic WS_2 over a range of wavelengths at little extra computational cost compared to single wavelengths.

Supplementary Note 2:

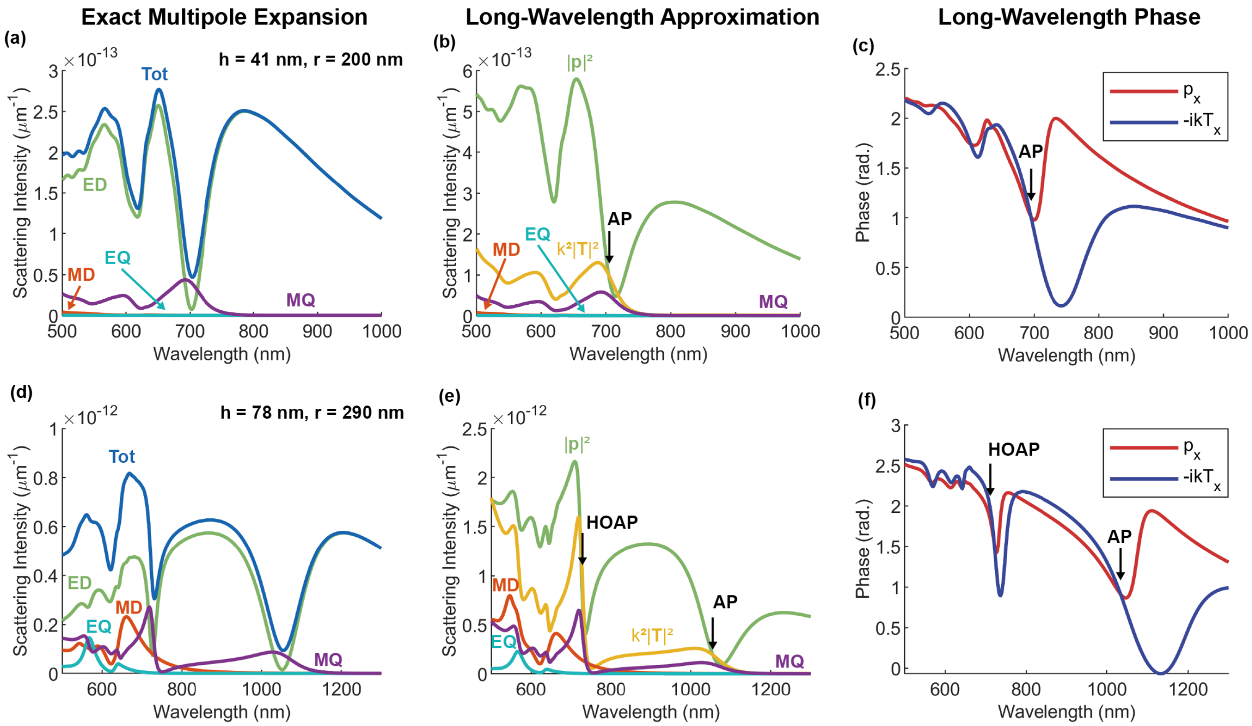

Figure S2: **Rigorous multipole expansion of Mie modes within WS_2 hexagonal nanoantennas in vacuum.** **(a)** Exact multipole expansion of Mie modes for a WS_2 nanoantenna of height 41 nm and radius 200 nm in vacuum. ED, MD, EQ, MQ, and Tot correspond to the scattering from the electric and magnetic dipoles, the electric and magnetic quadrupoles, and the sum of all contributions respectively. **(b)** Approximate multipole expansion where the ED mode has been decomposed into partial scattering cross-sections from the Cartesian electric dipole moment, $|\mathbf{p}|^2$, and toroidal electric dipole moment, $k^2|\mathbf{T}|^2$ where k is the wavenumber. AP corresponds to the anapole condition. **(c)** Phase calculation of electric (p_x) and toroidal ($-ikT_x$) dipole moments. **(d)**, **(e)**, and **(f)** show results for a nanoantenna of height 78 nm and radius 290 nm in vacuum for the same multipole expansion techniques. HOAP corresponds to the higher-order anapole.

In order to assign the contributions of individual Mie modes to peaks in the overall scattering spectra of WS_2 nanoantennas on gold, we must first understand the decomposition of the modes for the simpler case of a nanoantenna in vacuum. Here we employ the open source software MENP[2] in order to perform rigorous multipole expansions of the electric and magnetic fields within and around a WS_2 nanoantenna. We first demonstrate an exact multipole expansion[3] to identify the spectral positions and intensities of the base Mie modes

such as the electric dipole (ED), magnetic dipole (MD), electric quadrupole (EQ), and magnetic quadrupole (MQ) moments. The results of the exact solutions for the total and partial scattering based on the Mie model are shown in Figure S2(a) and (d) for two different sizes of nanoantennas.

To further identify the anapole states, we show the results for the long-wavelength approximation, where the electric dipole mode is decomposed into two terms following the method presented in Ref.,[2] i.e. $C_p = |\mathbf{p} + ik\mathbf{T}|^2$. Here C_p corresponds to the scattering cross-section from the electric dipole mode, $\mathbf{p}$ and $\mathbf{T}$ are commonly described as Cartesian and toroidal electric dipole moments respectively,[4] and k is the wavenumber. The partial scattering cross-sections associated with these two terms, denoted $|\mathbf{p}|^2$ and $k^2\,|\mathbf{T}|^2$ are shown in Figures S2(b) and (e) for nanoantennas of different sizes. As seen from the previous expression for C_p, a destructive interference condition is achieved when $\mathbf{p} = -ik\mathbf{T}$. We illustrate that this condition is achieved by showing that equal partial scattering cross-sections for $|\mathbf{p}|^2$ and $k^2\,|\mathbf{T}|^2$ in Figures S2(b) and (e) occur at the same wavelengths where the phases of the two contributions have the same magnitude and opposite signs as shown in Figure S2(c) and (f).

Results of the rigorous multipole expansion for the nanoantennas from Figures 1(d) and (e) of the main text are presented in Figure S2. For the smaller nanoantenna of height 41 nm and radius 200 nm, we see that the peaks in the total scattering are dominated almost entirely by the ED mode, with only a small contribution from the MQ mode at lower wavelengths (Figure S2(a)) causing the overall scattering to be non-zero. We can also identify the first dip at around 625 nm as absorption from the WS_2 exciton, which is present for all the spectra shown. Characterisation of the second dip at 700 nm requires use of the long-wavelength approximation and the decomposition of the electric dipole moment as shown in Figure S2(b). Importantly, we see a crossing of the partial scattering cross-sections from the $\mathbf{p}$ and $\mathbf{T}$ terms at 700 nm meaning that their respective scattering amplitudes are equal. This along with opposing phases as shown in Figure S2(c) confirm the anapole

condition at this wavelength.

We repeat the same procedure for the nanoantenna from Figure 1(e) of the main text with height 78 nm and radius 290 nm. Again we see that the ED mode dominates at higher wavelengths, however we see a small contribution from the MQ at around 1050 nm, and another at around 700 nm (Figure S2(d). In addition we now see contributions from the MD and EQ moments at around 650 and 560 nm respectively. Upon considering the decomposed ED mode as in Figure S2(e), we see two wavelengths where the **p** and **T** scattering cross-sections intersect. The phase plots in Figure S2 further confirm the anapole and higher-order anapole conditions at around 1040 nm and 720 nm, which we label as the AP and HOAP respectively.

With the constituent Mie modes and anapole states all identified for a WS_2 hexagonal nanoantenna in vacuum, we can now progress to moving the structure incrementally closer to a gold substrate as in Figures 1(d) and (e) of the main text. We are then able to directly visualise how each mode is affected by the hybridisation with gold, and how any new modes appear.

Supplementary Note 3:

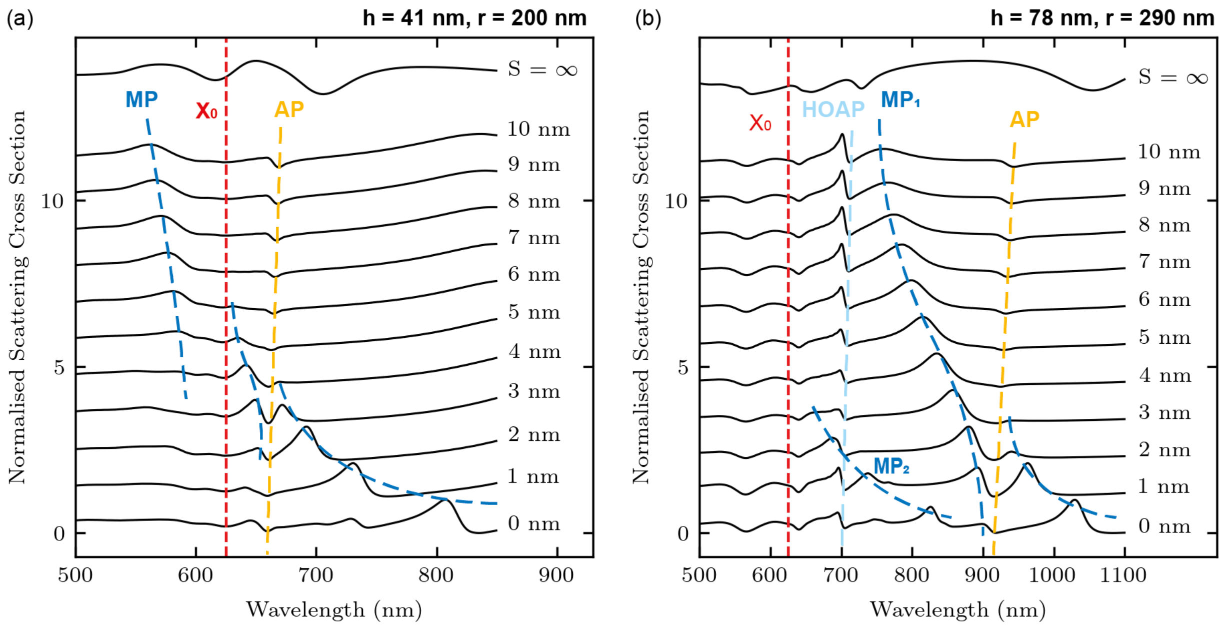

Figure S3: **Simulated scattering spectra with changing distance between a WS_2 nanoantenna and gold substrate, *s*. (a)**, **(b)** Enlarged scattering spectra from Figures 1(d) (h = 41 nm, r = 200 nm) and (e) (h = 78 nm, r = 290 nm) of the main text respectively for clearer visualisation of the mode evolution with the introduction of a gold substrate. MP, AP and HOAP correspond to the Mie-plasmonic mode, anapole, and higher-order anapole respectively. X_0 corresponds to the WS_2 exciton.

Here we show a smaller range of spectra from Figures 1(d) and (e) from the main text for $s =$ 0 - 10 nm. A reduced wavelength range is also considered in order to illustrate the behaviour of the MP modes in more detail. We observe an anti-crossing of the MP mode with the WS_2 exciton in Figure S3(a), as seen in previous studies of purely Mie modes.[5] In addition, we note further avoided crossings with the anapole for both nanoantenna geometries in Figures S3(a) and (b), which is a potential indication of strong mode coupling between the two resonances.

When s is reduced to 2 nm, another Fano-shaped peak emerges in Figure S3(b) at around 690 nm wavelength. At $s = 0$ nm the peak center shifts to 825 nm. This strong red-shift and Fano lineshape suggests that higher-order MP modes can exist within WS_2 nanoantennas on a gold substrate hence the naming MP_1 and MP_2.

Supplementary Note 4:

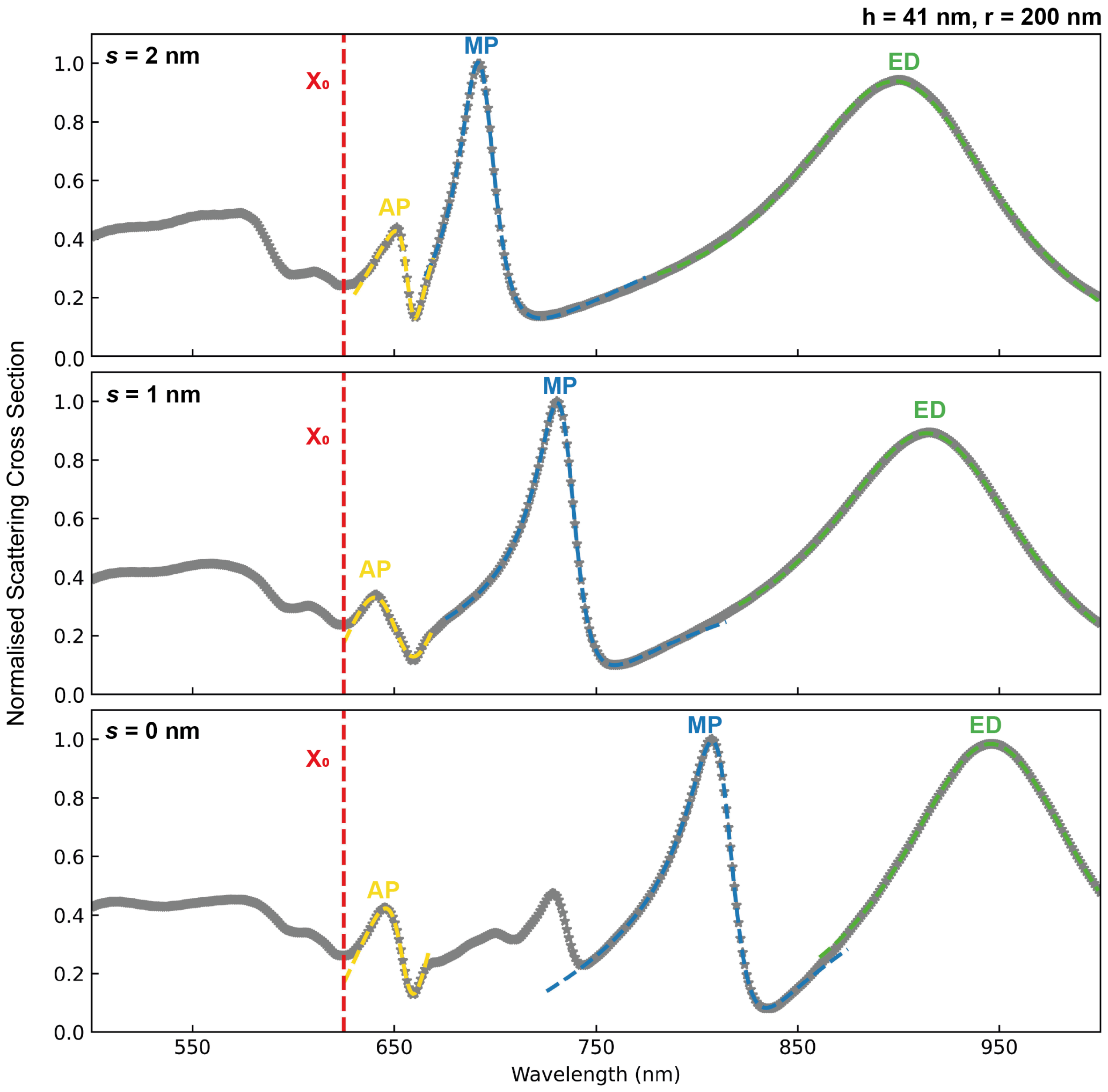

Figure S4: **Example curve fitting to simulated scattering spectra of WS_2 nanoantennas on gold.** Nanoantenna of height 41 nm and radius 200 nm considered with distances from the gold substrate $s = 0$, 1, and 2 nm (bottom, middle, and top panels respectively). Grey stars correspond to simulated data points using the FDTD method. Dashed lines labelled AP, MP, and ED correspond to fits to the anapole, Mie-plasmonic, and electric dipole modes respectively. X_0 denotes the position of the WS_2 exciton.

In Figure S4, we show three examples of normalised scattering cross-sections obtained via FDTD simulation of a WS_2 nanoantenna on/above gold from Figure 1(d) of the main text. The value s corresponds to the distance between the bottom surface of the nanoantenna and top surface of the gold substrate. Overlaid with dashed lines, are fits to the various modes we study in the main text. For the anapole and Mie-plasmonic mode, we use a Fano formula to account for the interaction of the discrete Mie modes with the plasmon continuum. In contrast, we fit the electric dipole mode with a Lorentzian peak owing to the negligible effect of plasmons for this resonance. From such fits we are able to extract peak centre positions and linewidths, hence allowing us to calculate respective quality factors by dividing the two values.

Supplementary Note 5:

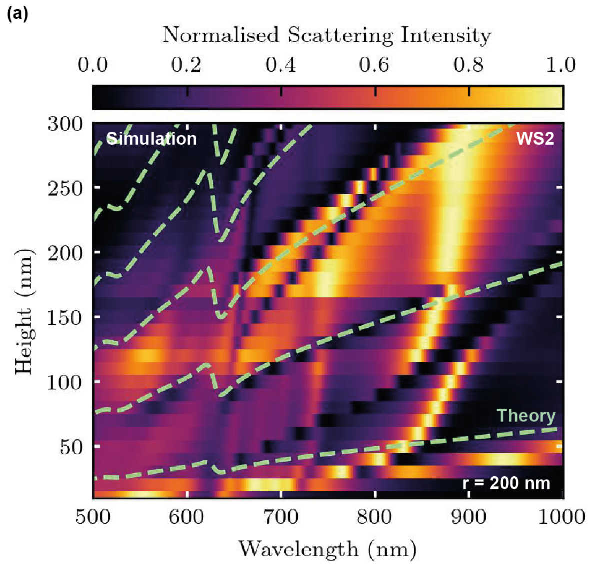

Figure S5: **Comparison of Fabry–Perot mode theory with FDTD data for hexagonal monomers of varying height on a gold substrate.** **(a)**, **(b)** correspond to monomers using the refractive index of WS_2, and a constant refractive index of 4.1 respectively. All radii are set to 200 nm. Light green dashed lines correspond to predicted FP mode positions from theory.

In order to host a Fabry–Perot (FP) resonance within a cavity, the round-trip phase accumulation of a wave within it must equal an integer number (m) of 2π radians. If we consider our cavity to be a dielectric nanoantenna on a gold substrate, we arrive at the following equation

$$2\beta H + \phi_b + \phi_t = 2m\pi, \tag{S1}$$

where β is the phase constant of the wave in a nanoantenna of height H, and $\phi_{b,t}$ are the phase changes upon reflection from the bottom and top surfaces of the nanoantenna respectively. We assume the gold to be a perfect electrical conductor, and hence the reflection phase change at the gold-WS_2 boundary is π. In contrast, $\phi_t = 0$ as the wave reflects from a boundary where the outside (i.e. vacuum) is of a lower refractive index than within the nanoantenna. The phase constant can then be calculated from

$$\beta = \omega \sqrt{\frac{\mu\epsilon}{2}\left(\sqrt{1+\left(\frac{\sigma}{\omega\epsilon}\right)^2}+1\right)} \tag{S2}$$

where w is the vacuum angular frequency, μ and ϵ are the absolute permeability and permittivity of WS_2 respectively, and σ is its electrical conductivity. By then setting H in Equation S1, we can predict the wavelengths at which the total round-trip phase equals $2m\pi$, as shown in Figure S5.

We see good agreement of our FP mode model (dashed green lines in Figure S5) with the dark modes in our FDTD simulations for both WS_2, and constant refractive index (n = 4.1) monomers on gold. For the n = 4.1 monomers, the FP modes red-shift linearly with height in the scattering spectra, in agreement with theory. When WS_2 is considered in Figure S5(a), the exciton at 625 nm causes many of the modes to become unresolvable. However, at larger wavelengths the mode also red-shifts linearly with increasing nanoantenna height. This is predicted by theory well, where we see an asymmetric feature around the excitonic absorption, followed by a linear increase in wavelength with nanoantenna height. Notably, the modes in the scattering spectra are blue-shifted compared to the theory. This can likely be attributed to the lateral confinement of the FP mode owing to the finite size of the nanoantennas. The FP mode model used considers an infinite plane dielectric, and so does not take into account edge effects introduced by the nanoantennas.

Supplementary Note 6:

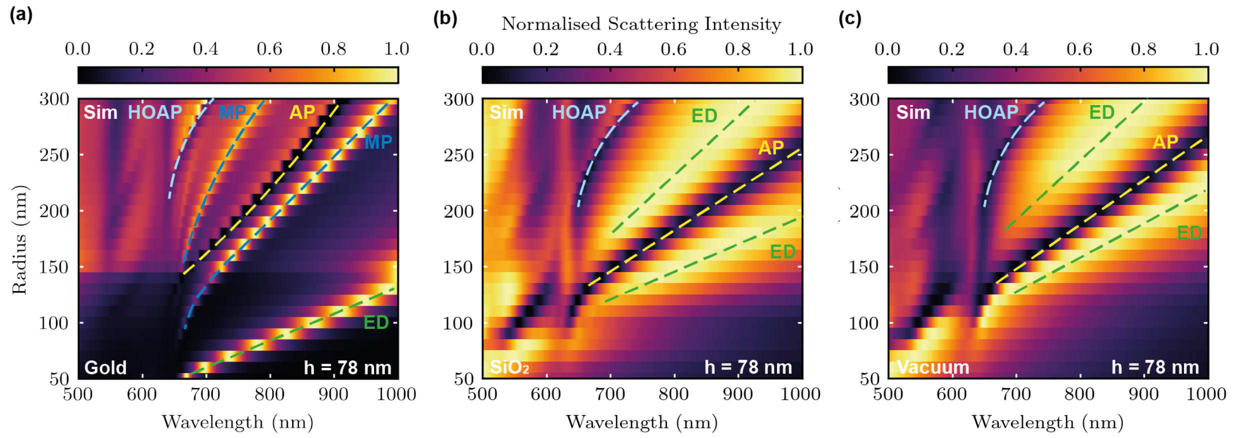

Figure S6: **Normalised scattering intensity for WS_2 hexagonal monomer nanoantennas of height 78 nm with different substrates in simulation.** **(a)**, **(b)**, and **(c)** correspond to gold, SiO_2 and vacuum substrates respectively, for the same range of radii. ED corresponds to the electric dipole mode, MP corresponds to Mie-plasmonic mode, and AP and HOAP stand for anapole and higher-order anapole respectively.

As shown in Figure S6, we expect a vastly differently mode structure for WS_2 nanoantennas placed on a gold substrate compared to that on a SiO_2 substrate. The bright modes are much narrower when using gold, suggesting higher Q factor resonances. In addition, the dark mode (i.e. the anapole) exhibits a Fano lineshape when using a gold substrate, but is described by a Lorentzian in the SiO_2 and vacuum cases. The Fano curve suggests an interference of a discrete state and a continuum (Mie mode and plasmons), which is further reinforced by the fact that we do not see this lineshape with a dielectric substrate. Another point to note is that the AP and HOAP modes are blue-shifted when using a gold substrate compared to SiO_2. This suggests a confinement of the modes, likely due to the gold substrate which reflects much of the light back into the nanoantenna unlike SiO_2. In contrast, the ED mode red-shifts with the introduction of a gold substrate which can be explained by the increased mode volume seen in Figure 3(d) of the main text. WS_2 nanoantennas in vacuum (Figure S6(c)) are shown as a comparison. The scattering intensity is very similar to that of the case with a SiO_2 substrate, owing to its low refractive index throughout the visible

wavelength range.[6]

Supplementary Note 7:

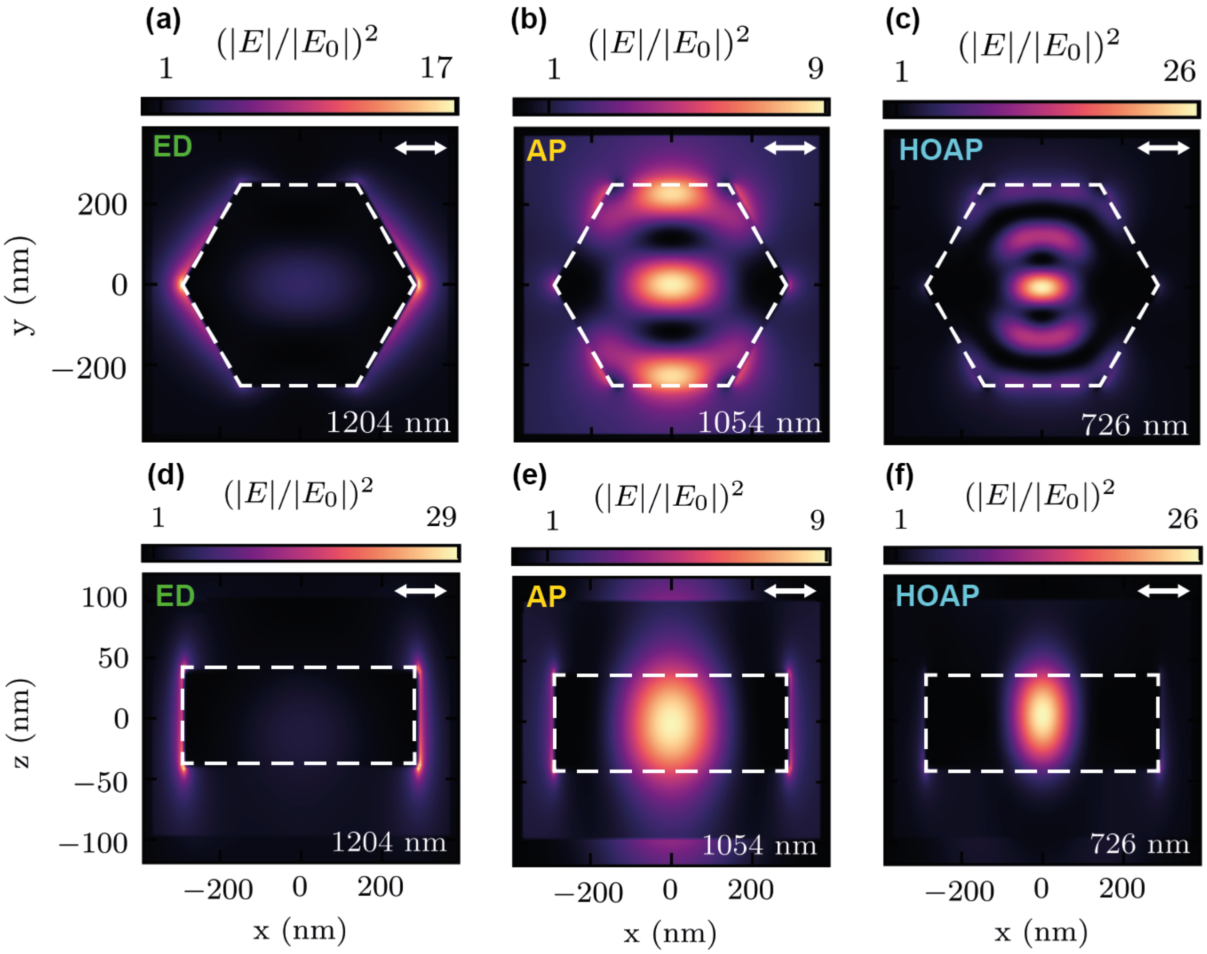

Figure S7: **Simulated electric field intensity within a WS_2 nanoantenna of height 78 nm and radius 290 nm in vacuum for different resonant Mie modes.** **(a)**, **(b)**, and **(c)**, correspond to the electric fields at the electric dipole mode, anapole, and higher-order anapole in a cross-section through the middle of the nanoantenna in the xy plane respectively. **(d)**, **(e)**, and **(f)** correspond to the same modes in the xz plane. Incident plane wave is polarised along the x axis. Bottom right value in each panel denotes incident wavelength. White dashed boxes indicate the nanoantenna edges.

The electric dipole mode for a hexagonal WS_2 nanoantenna suspended in vacuum is confined most strongly at and around the vertices,[7] along the polarisation direction of the incoming light, as shown in Figures S7(a) and (d). A central lobe is present within the nanoantenna, however the electric field intensity is much weaker than at the vertices. In Figures S7(b) and (e), we note that the anapole resonance is strongly confined to within the nanoantenna

boundary, with very little leakage to the environment. This is also true for the HOAP, with both resonances possessing high quality factors owing to the strong confinement. These field distributions serve as a comparison to those shown in Figures 3(a)-(f) for WS_2 nanoantennas on a gold substrate. Since there is no substrate in Figure S7, we observe that the electric field lobes are mostly symmetric about the z = 0 plane, extending equally out of the top and bottom of the nanoantenna structure.

Supplementary Note 8:

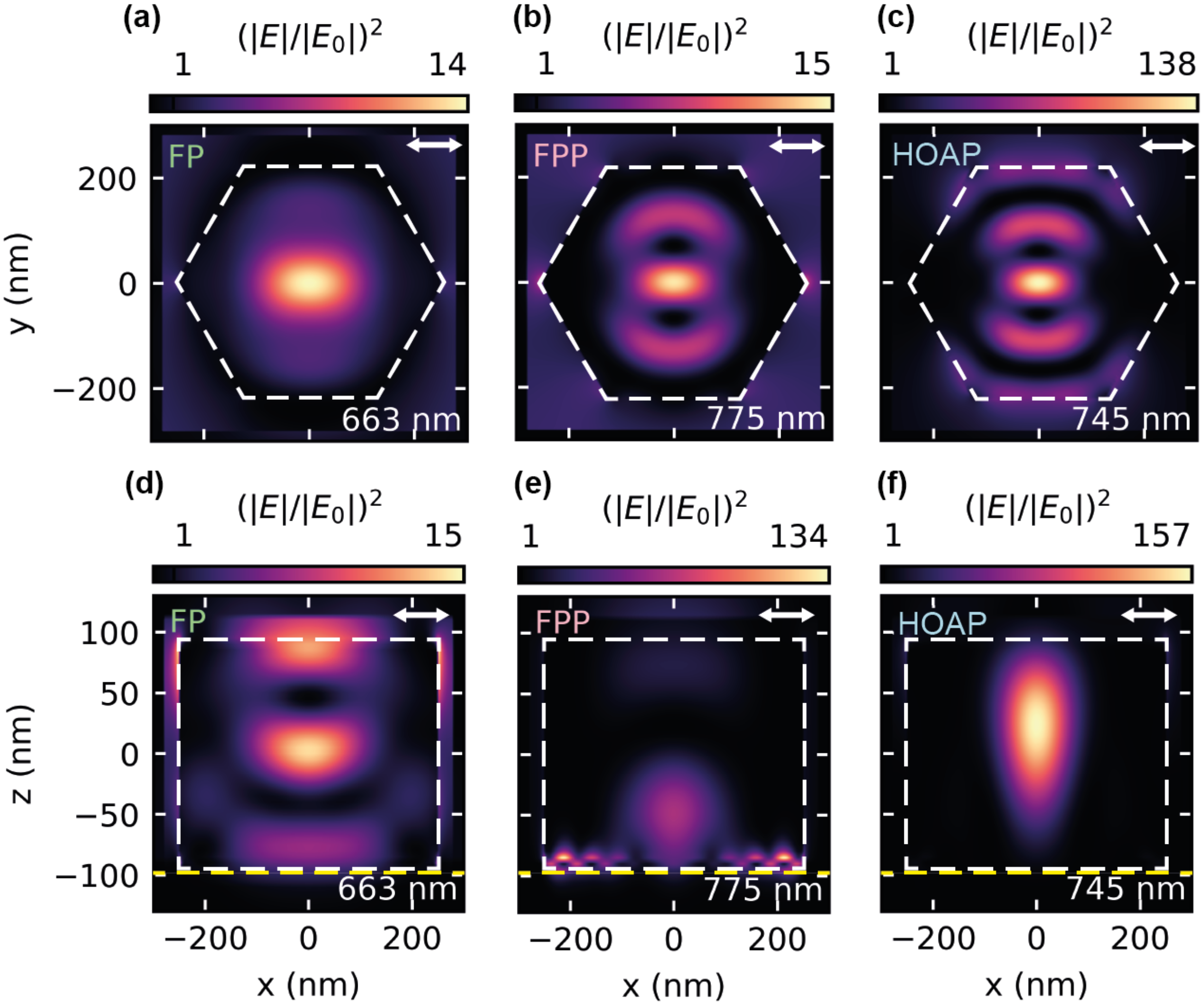

Figure S8: **Simulated electric field intensity within a WS_2 nanoantenna of height 180 nm and radius 250 nm on a gold substrate for different hybrid resonances.** **(a)**, **(b)**, and **(c)**, correspond to the electric fields for the Fabry–Perot mode, Fabry–Perot-plasmonic mode, and higher-order anapole in a cross-section through the middle of the nanoantenna in the xy plane respectively. **(d)**, **(e)**, and **(f)** correspond to the same modes in the xz plane. Incident plane wave is polarised along the x axis. Bottom right value in each panel denotes incident wavelength. White dashed boxes indicate the nanoantenna edges, and gold dashed line represents the position of the gold substrate

In Figure S8 we show electric field distributions of the Fabry–Perot (FP) mode, Fabry–Perot-plasmonic (FPP) mode, and higher-order anapole state (HOAP) in the WS_2 nanoantenna on gold structure from Figure 2(c) and (f) of the main text. Owing to the reflections

introduced by the gold substrate, vertically propagating Fabry–Perot-type modes can be realised as seen by the three vertically-arranged maxima in Figure S8(d). In addition, since there is a π phase shift at the gold-TMD boundary, the effective mode volume is doubled and larger wavelengths of light can be confined within the same sized structure compared to a nanoantenna on a SiO_2 substrate for example. The gold-TMD boundary also enables the necessary k-space matching allowing for the formation of plasmons at certain wavelengths of incident light. In Figure S8(e) we observe two vertically-stacked maxima, similar to in Figure S8(d), but also a strong confinement of light at the gold-TMD boundary, suggesting a hybridisation of a Fabry–Perot mode with plasmons. This mode also take a Fano lineshape in the spectra as opposed to the Lorentzian lineshape of the FP mode, further confirming a hybridisation with plasmons for the FPP mode. Finally, we observe that the HOAP for a nanoantenna on gold (Figure S8(c) and (f)) possesses a very similar field distribution to that of a nanoantenna suspended in vacuum (Figure S7(c) and (f)). The main differences being an order of magnitude higher intensity owing to the confinement from the gold substrate, and a warped, asymmetric field maxima in the xz plane likely owing to the interaction of the mode with it's mirror image.

Note that the field distribution in the xy plane for the FPP mode in Figure S8(b) appears similar to that of the HOAP in Figure S8(c). However, the intensity of the FPP field is an order of magnitude lower than that of the HOAP, and the field distribution within the xz plane in Figure S8(e) is significantly different to that of the HOAP in Figure S8(f). This similarity in the xy plane is therefore likely due to the spectral proximity of this FPP mode to the HOAP ($\sim$ 30 nm) causing some preliminary hybridisation. Both modes can be tuned to fully hybridise by varying the nanoantenna radius as demonstrated in Figure 4(a) and (c) of the main text.

Supplementary Note 9:

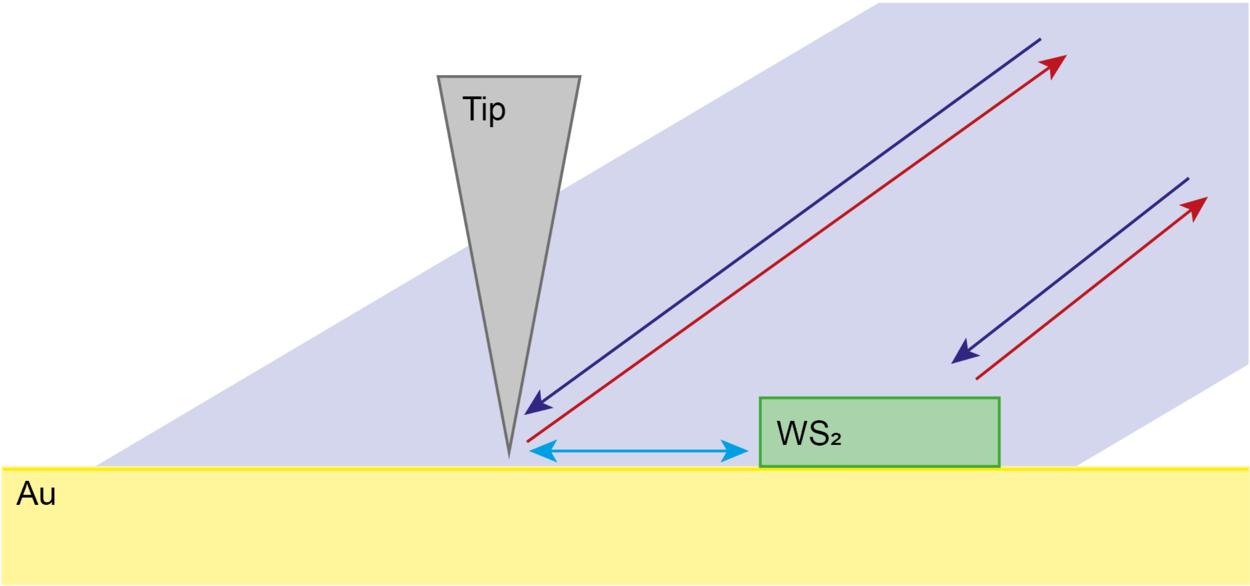

Figure S9: **Schematic of the tip-nanoantenna interaction region during scattering-type scanning near-field optical microscopy (s-SNOM).** Dark blue arrows correspond to light incident on the tip and WS_2 nanoantenna. Red arrows correspond to light scattered from the tip and nanoantenna. Cyan double-headed arrow represents the interaction between the nanoantenna and tip.

s-SNOM involves probing the near-field response of a sample with an illuminated AFM tip, as illustrated schematically in Figure S9, and using interferometric techniques to resolve both the amplitude and phase of the light scattered from the tip-sample interaction region. The tip can then be scanned across the sample, as in Figure 3(g) of the main text, which shows the amplitude of the light scattered from the tip-sample interaction at each point. This measured value contains information correlated to both the material permittivities, as well as any electric fields probed by the tip. For example, we observe the formation of ripples around the nanoantennas, which can be attributed to the tip illumination source interfering with SPPs on the gold, or with scattered light from features on the sample.[8–13]

As the tip is moved across the sample, the contributions to the s-SNOM signal either interfere constructively or destructively. This produces a pattern of bright and dark fringes in the amplitude of the scattered light, which is observed as ripples. There are several methods

in which sample-scattered light and SPPs can interact with the incident light at the tip, and each produce different interference patterns.[8,9,12] For the case of WS_2 nanoantennas on gold however, we consider two prominent mechanisms, the first being tip-launched SPPs. As the incident light reaches the tip (left dark blue arrow in Figure S9), it becomes strongly localised at the tip's apex. This strong near-field enhancement, and matching of the photon and plasmon wavevectors, causes tip-launched plasmons that emanate radially into the gold.[14] Such SPPs can then reflect from nearby structures and interfere with the incident light back at the tip as depicted by the double-headed cyan arrow in Figure S9. The second mechanism involves SPPs launched from the nanoantennas.[14] The right dark blue arrow in Figure S9 represents light incident on the nanoantenna, which causes subsequent excitation of a resonance, and launching of SPPs which travel to the tip and interfere with the incoming light (cyan arrow). A combination of these two effects is observed in the ripple patterns in Figure 3(g) of the main text.

We note that there are several further mechanisms that may contribute to the interference patterns observed in Figure 3(g) of the main text, such as photon scatter from the nanoantennas, s-SNOM tip, and sample substrate, as well as a host of second- and higher-order mechanisms. However, given the correlation between the amplitude of the s-SNOM interference ripples and the dark field scattering data associated with individual nanoantennas (as discussed in the main text and below in Supplementary Note 10), alongside the data presented in Supplementary Note 12 of s-SNOM of low-index materials on gold, we suggest that the mechanisms discussed above describe the data appropriately.

Supplementary Note 10:

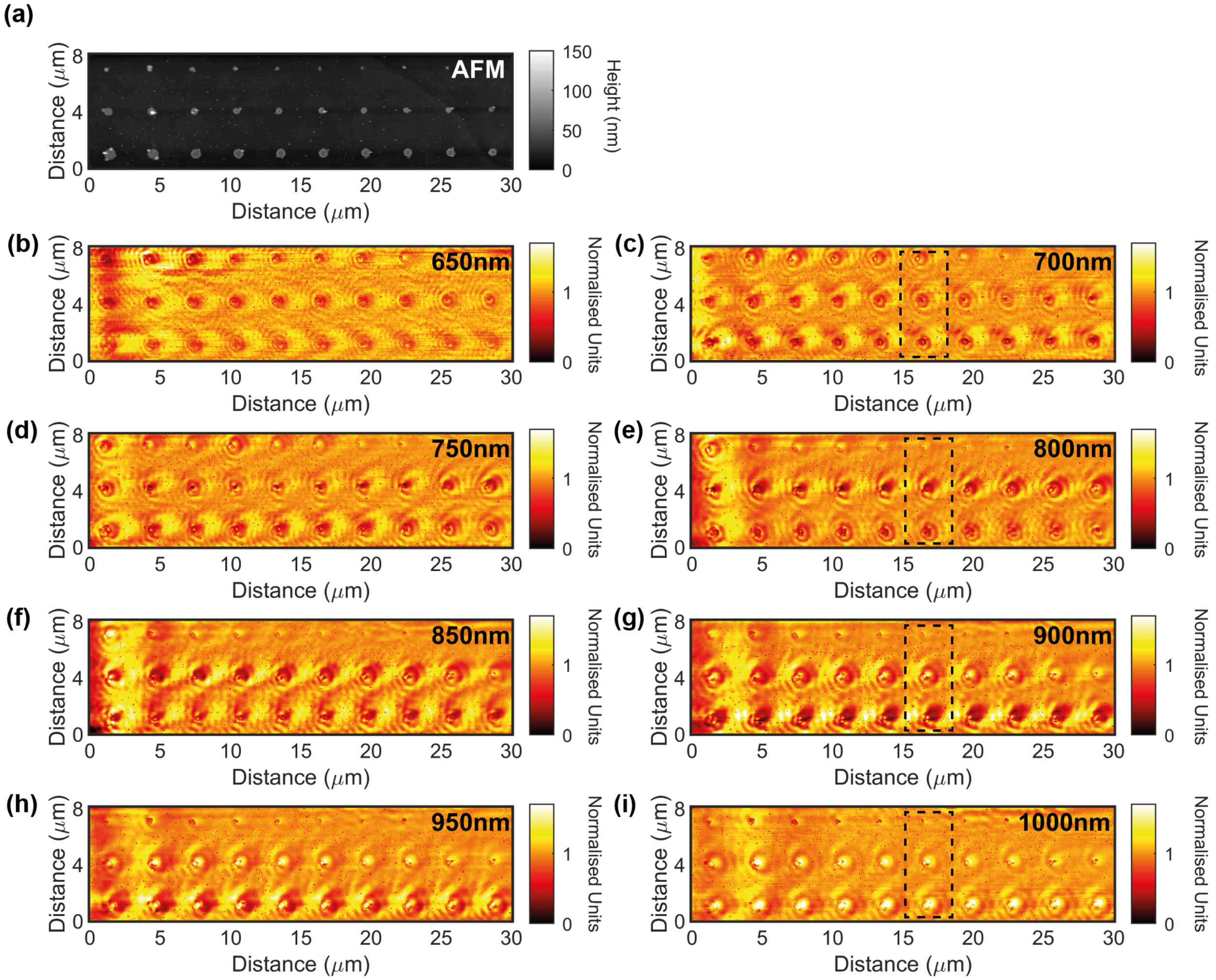

Figure S10: **Additional experimental s-SNOM amplitude data of a full array of WS_2 nanoantennas of height 41 nm and increasing radii on gold at different excitation wavelengths.** **(a)** AFM topography image of the array of nanoantennas. **(b)-(i)** correspond to s-SNOM amplitude images at 650 to 1000 nm excitation in 50 nm increments. Radii increase in roughly 10 nm increments from the top right to the bottom left of the array. Black dashed boxes indicate the nanoantennas studied in Figure 3(g) of the main text.

We recorded the scattering amplitude from s-SNOM of WS_2 nanoantennas of height 41 nm and varying radii at a range of excitation wavelengths for comparison. As the incident wavelength is varied, the wavelength of the SPPs changes according to their dispersion relation, resulting in a change of the distance between the maxima of the ripple intensities. From Figure S10, we note that the distance between the ripple maxima increases with the wavelength

of the excitation laser, but is constant for nanoantennas with different radii. Most notably however, we observe that the intensity of the SPP ripples follows the red-shift of Mie modes of the nanoantennas with increasing radius. This can be seen by comparing the position of the peaks in the dark field spectra of individual nanoantennas from Figure 3(h) of the main text, with their respective s-SNOM images (dashed boxes in Figures S10(c), (e), (g), and (i)) at different wavelengths. The observation of the intensity of SPP interference patterns being correlated to the spectral positions of the Mie modes is true for all 30 nanoantennas, which gives further evidence to support the idea of hybridised Mie-plasmonic modes which can launch and enhance SPPs from dielectric nanoantennas on gold.

Supplementary Note 11:

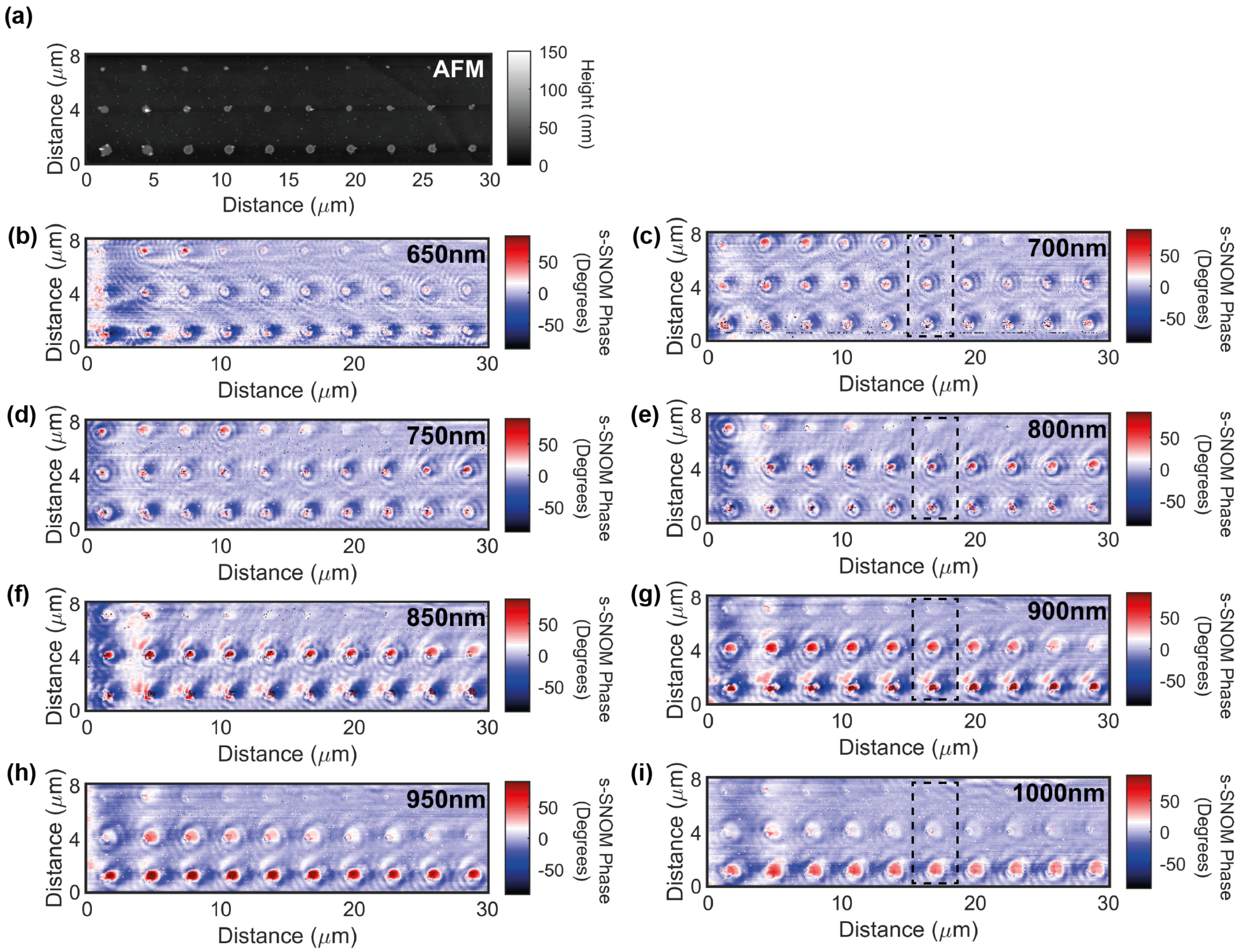

Figure S11: **Additional experimental s-SNOM phase data of a full array of WS_2 nanoantennas of height 41 nm and increasing radii on gold at different excitation wavelengths.** **(a)** AFM topography image of the array of nanoantennas. **(b)-(i)** correspond to s-SNOM phase images at 650 to 1000 nm excitation in 50 nm increments. Radii increase in roughly 10 nm increments from the top right to the bottom left of the array. Black dashed boxes indicate the nanoantennas shown in Figure 3(g) of the main text.

We present the decoupled scattering phase data of our s-SNOM measurements from Figure S10 by using the pseudo-heterodyne detection technique as described in the methods section of the main text. Each line in every image is median levelled to account for thermal drift, then added to the median of the entire image to give a relative phase. Importantly, we note that all the ripple patterns observed in the scattering data are reproduced in the phase data

with the same relative intensities, as are the wavelengths of the patterns.

Supplementary Note 12:

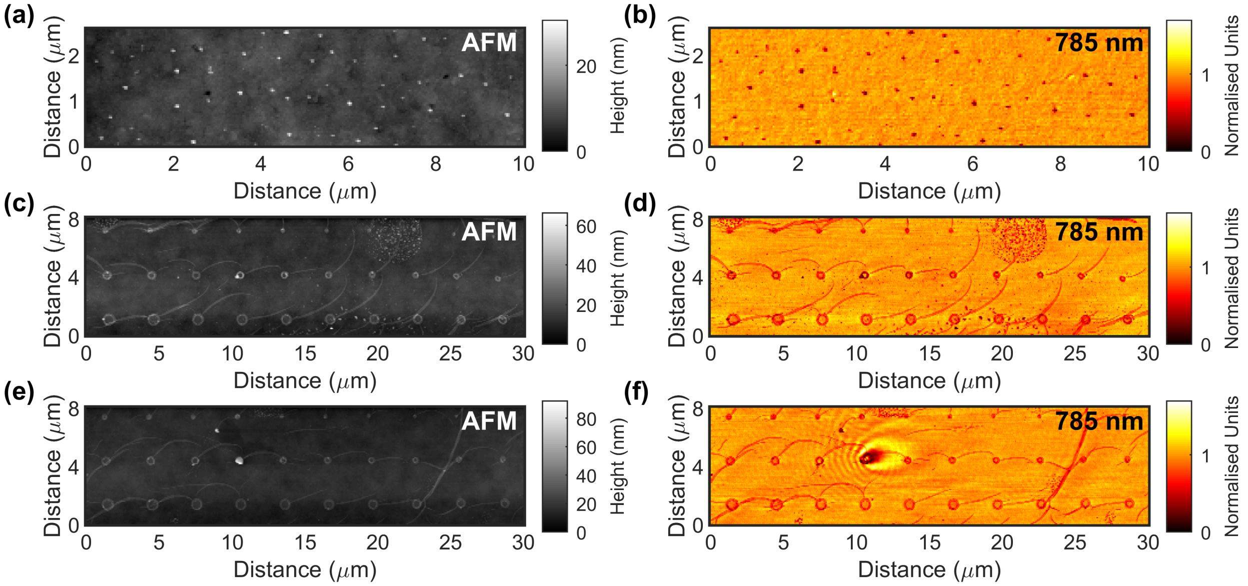

Figure S12: **AFM and s-SNOM images of gold substrate and resist pillars on a gold substrate at 785 nm excitation.** **(a)** and **(b)** correspond to AFM and s-SNOM images of the gold substrate away from any nanoantennas respectively. **(c)** and **(d)** correspond to AFM and s-SNOM images of resist pillars on gold of comparable height to the WS_2 nanoantennas measured in Figure 3(g) of the main text respectively. **(e)** and **(f)** correspond to AFM and s-SNOM respectively of additional resist pillars on gold with a single WS_2 nanoantenna remaining, surrounded by an SPP interference pattern.

Figure S12(b) shows s-SNOM imaging of an area of gold substrate away from the WS_2 nanoantennas yielding no SPP ripple pattern as expected. Tip-launched plasmons are still present in this case, however they have no large structures nearby to reflect back from and therefore do not interference with the tip-incident light. When we introduce pillars of resist of comparable height to the WS_2 nanoantennas measured in Figure 3(k), we do not observe strong SPP patterns in the s-SNOM image (Figure S12(d)). The resist pillars do not host strong Mie resonances at visible wavelengths owing to their low refractive indices (1.49[15]), and so coupling to plasmons is weak. Furthermore, tip-launched plasmons are expected to transmit through such structures with very little reflection back to the tip. In Figure S12(f), we image an array of resist pillars with a single WS_2 nanoantenna in the middle. Strong SPP ripples are observed around the nanoantenna, suggesting that Mie resonances inside

the structure hybridise with plasmonic resonances to enhance radially-launched SPPs. The SPP pattern is not circular in this case, owing to the incident light scattering from plasmons interacting with the tip.[8] This observation further implies that only the high refractive index, WS_2 nanoantennas can launch SPPs through coupling with their photonic resonances.

Supplementary Note 13:

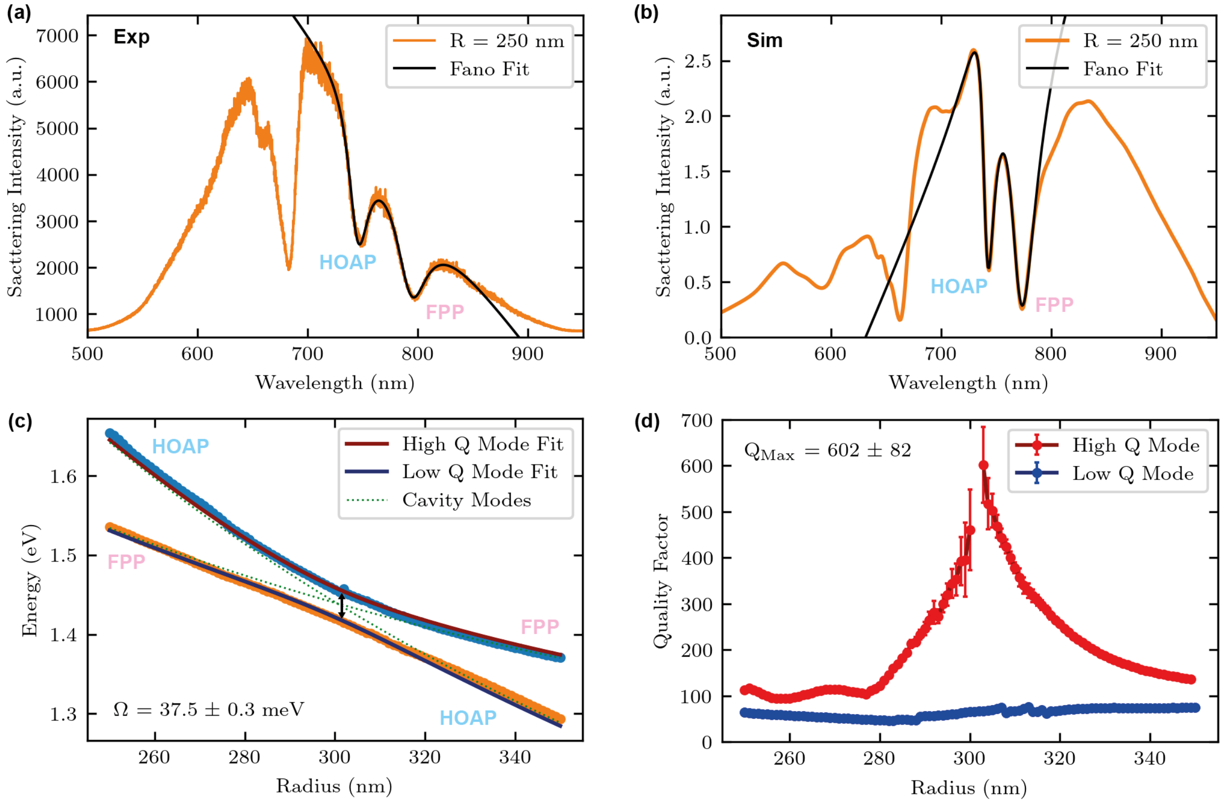

Figure S13: **Fano fits to simulated and experimental scattering spectra from WS_2 nanoantennas on a gold substrate.** **(a)** and **(b)** correspond to individual scattering spectra from dimer nanoantennas of height 180 nm and radius 250 nm from experiment and simulation respectively (orange lines). Black lines show double Fano curve fits to the higher-order anapole (HOAP) and Fabry–Perot-plasmonic (FPP) mode. **(c)** Simulated peak positions of the HOAP and FPP mode fitted to a coupled oscillator model, from optimised nanoantennas of 200 nm height and varying radii. Ω denotes the minimum energy splitting between the modes. **(d)** Extracted quality factor for all peaks fitted in **(c)**.

In order to fit the anti-crossing observed between the higher-order anapole (HOAP) and Fabry–Perot-plasmonic (FPP) mode in Figures 2(c) and (f) of the main text, we used a coupled oscillator model. To do this, we first fitted each peak corresponding to the HOAP and FPP mode in both the simulated and experimental scattering spectra of WS_2 nanoantennas on gold to a double Fano curve. Examples of individual fits for single nanoantennas from both experiment and simulation are shown in Figures S13(a) and (b) respectively, corresponding

to a height of 180 nm and a radius of 250 nm. This type of fit was chosen owing to the hybrid Mie-plasmonic nature of each of the modes, where we observe an interference between a resonant state and a continuum of states. To account for the two peaks, a double Fano formula was used as in Equation S3,

$$y = mx + y_0 - |A_l| \frac{(q_l + \epsilon_l)^2}{1 + \epsilon_l^2} - |A_u| \frac{(q_u + \epsilon_u)^2}{1 + \epsilon_u^2} \tag{S3}$$

where

$$\epsilon_l = \frac{x - x_{lc}}{\Gamma_l}, \quad \epsilon_u = \frac{x - x_{uc}}{\Gamma_u} \tag{S4}$$

and $A_{l,u},\ q_{l,u},\ x_{l,u},\ \Gamma_{l,u}$ are the relative amplitudes, asymmetry parameters, peak center positions, and full-width-at-half-maxima of the lower and upper wavelength peaks respectively. The double Fano curves are overlaid in Figures S13(a) and (b) in black, and show good agreement with both the experimental and simulated data. We repeated this fitting process for all of the fabricated nanoantennas of height 180 nm with radii ranging from 220 to 330 nm in, on average, 10 nm increments. The peak center positions were then fitted to a coupled oscillator model as shown in Figure 4(a) of the main text. We then simulated the same sized nanoantennas but with a finer step in radius of 1 nm. We encountered difficulties in fitting owing to a broader resonance in close spectral proximity to the anti-crossing, and so optimised the nanoantenna height in order to red-shift the anti-crossing sufficiently far from the broad peak. Dimer nanoantennas of height 200 nm and radii ranging from 250 to 350 nm were then simulated. The scattering spectra were fitted using the same process as with the experimental data giving the plot in Figure S13(c), where the theoretical uncoupled HOAP and FPP mode are represented by the dotted green lines labelled cavity modes. We observe a distinct anti-crossing of the two modes, and fit them to a coupled oscillator model, yielding an upper and lower energy branch depicted by red and blue lines respectively. We refer to such lines as the high and low Q factor modes respectively, owing to their respective

quality factors in the scattering spectra. From this fitting, we extract an energy splitting Ω of 37.5 $\pm$ 0.3 meV, which is greater than the sum of the half linewidths of the HOAP and FPP mode away from the closest point to the anti-crossing, hence confirming strong mode coupling.

Furthermore, upon plotting the Q factors of each of the branches against nanoantenna radius as in Figure S13(d), we see that the low Q factor mode remains approximately constant, whereas the high Q factor mode peaks significantly for a radius of 302 nm. We calculate a maximum Q factor of 602 $\pm$ 82 for a nanoantenna radius of 303 nm, with the high error owing to the suppression of the peak in the scattering spectra. This can be seen in Figure 4(d) of the main text, where the high Q peak becomes difficult to fit in the range 301 - 303 nm as a result of the destructive interference between the two photonic modes. This behaviour is a signature of a Friedrich-Wintgen bound state in the continuum (BIC),[16] and leads to a highly confined mode with a theoretically infinite Q factor for a nanoantenna with radius corresponding to the lowest energy splitting between the modes. This is supported by our results in Figure S13(d), which shows an exponentially increasing Q factor for a radius of 302 nm, which is also the closest point to the anti-crossing in Figure S13(c). Since an infinite Q factor is, by nature, impossible to measure; we attribute this mode to a quasi-BIC from our simulated data. In the context of finite-sized nanoantennas, we term this a supercavity mode.[17]

References

(1) Zotev, P. G.; Wang, Y.; Andres-Penares, D.; Severs-Millard, T.; Randerson, S.; Hu, X.; Sortino, L.; Louca, C.; Brotons-Gisbert, M.; Huq, T.; others Van der Waals materials for applications in nanophotonics. *Laser & Photonics Reviews* **2023**, 2200957.

(2) Hinamoto, T.; Fujii, M. MENP: An open-source MATLAB implementation of multipole expansion for nanophotonics. *OSA Continuum* **2021**, *4*, 1640–1648.

(3) Alaee, R.; Rockstuhl, C.; Fernandez-Corbaton, I. An electromagnetic multipole expansion beyond the long-wavelength approximation. *Optics Communications* **2018**, *407*, 17–21.

(4) Baryshnikova, K. V.; Smirnova, D. A.; Luk'yanchuk, B. S.; Kivshar, Y. S. Optical anapoles: concepts and applications. *Advanced Optical Materials* **2019**, *7*, 1801350.

(5) Verre, R.; Baranov, D. G.; Munkhbat, B.; Cuadra, J.; Käll, M.; Shegai, T. Transition metal dichalcogenide nanodisks as high-index dielectric Mie nanoresonators. *Nature Nanotechnology* **2019**, *14*, 679–683.

(6) Rodríguez-de Marcos, L. V.; Larruquert, J. I.; Méndez, J. A.; Aznárez, J. A. Self-consistent optical constants of SiO_2 and Ta_2O_5 films. *Optical Materials Express* **2016**, *6*, 3622–3637.

(7) Choi, H.; Heuck, M.; Englund, D. Self-similar nanocavity design with ultrasmall mode volume for single-photon nonlinearities. *Physical Review Letters* **2017**, *118*, 223605.

(8) Chang, Y.-C.; Chu, J.; Wang, T.; Lin, M.; Yeh, J.; Wang, J.-K. Fourier analysis of surface plasmon waves launched from single nanohole and nanohole arrays: unraveling tip-induced effects. *Optics express* **2008**, *16*, 740–747.

(9) Bozhevolnyi, S. I. Near-field mapping of surface polariton fields. *Journal of Microscopy* **2001**, *202*, 313–319.

(10) Kaltenecker, K. J.; Krauss, E.; Casses, L.; Geisler, M.; Hecht, B.; Mortensen, N. A.; Jepsen, P. U.; Stenger, N. Mono-crystalline gold platelets: A high-quality platform for surface plasmon polaritons. *Nanophotonics* **2020**, *9*, 509–522.

(11) Zhang, C.; Hugonin, J.-P.; Greffet, J.-J.; Sauvan, C. Surface plasmon polaritons emission with nanopatch antennas: Enhancement by means of mode hybridization. *ACS Photonics* **2019**, *6*, 2788–2796.

(12) Walla, F.; Wiecha, M. M.; Mecklenbeck, N.; Beldi, S.; Keilmann, F.; Thomson, M. D.; Roskos, H. G. Anisotropic excitation of surface plasmon polaritons on a metal film by a scattering-type scanning near-field microscope with a non-rotationally-symmetric probe tip. *Nanophotonics* **2018**, *7*, 269–276.

(13) Li, Y.; Zhou, N.; Kinzel, E. C.; Ren, X.; Xu, X. The origin of interferometric effect involving surface plasmon polariton in scattering near-field scanning optical microscopy. *Optics express* **2014**, *22*, 2965–2972.

(14) Novotny, L.; Hecht, B. *Principles of Nano-Optics*; Cambridge University Press: Cambridge, 2012.

(15) Zhang, X.; Qiu, J.; Li, X.; Zhao, J.; Liu, L. Complex refractive indices measurements of polymers in visible and near-infrared bands. *Applied Optics* **2020**, *59*, 2337–2344.

(16) Friedrich, H.; Wintgen, D. Interfering resonances and bound states in the continuum. *Physical Review A* **1985**, *32*, 3231.

(17) Rybin, M. V.; Koshelev, K. L.; Sadrieva, Z. F.; Samusev, K. B.; Bogdanov, A. A.; Limonov, M. F.; Kivshar, Y. S. High-Q supercavity modes in subwavelength dielectric resonators. *Physical Review Letters* **2017**, *119*, 243901.
